# Efficacy and safety of oral proprietary Chinese medicines in the treatment of stable chronic obstructive pulmonary disease: a network meta-analysis

**DOI:** 10.3389/fphar.2025.1690739

**Published:** 2026-01-21

**Authors:** Hao Yan, Jingwen Zhang, Hutao Yan, Dandan Yang

**Affiliations:** 1 Traditional Chinese Medicine Department, The Third People’s Hospital of Henan Province, Zhengzhou, Henan, China; 2 Traditional Chinese Medicine Department, The Third People’s Hospital of Henan Province-Longhu Town Wenchang Road Community Health Service Center, Zhengzhou, Henan, China; 3 Hospital Pharmacy, The Third People’s Hospital of Henan Province, Zhengzhou, Henan, China

**Keywords:** chronic obstructive pulmonary disease, efficacy, network meta-analysis, oral proprietary Chinese medicine, safety

## Abstract

**Background:**

A variety of oral proprietary Chinese medicines (OPCMs) have clinical efficacy in the adjunctive treatment of stable chronic obstructive pulmonary disease (COPD). However, the OPCM with the best therapeutic effect is not yet clear. Thus, a network meta-analysis (NMA) is leveraged to evaluate the best efficacious OPCM for the adjunctive treatment of stable COPD.

**Methods:**

Randomized controlled trials (RCTs) related to the adjunctive treatment of stable COPD with OPCMs were searched in PubMed, Web of Science, Embase, Cochrane Library, China National Knowledge Infrastructure, Wanfang, and VIP. The search period was up to 1 April 2024. Study screening and data extraction were performed according to predefined inclusion and exclusion criteria. The assessment of bias in the included studies was carried out using the Cochrane risk of bias tool version 2 (RoB 2.0). Statistical analyses were performed utilizing Stata version 17.0 (64-bit) and R software (version 4.3.3).

**Results:**

The database retrieval yielded 7,572 articles in total. Ultimately, 64 articles were included in the analysis. Compared to routine treatment (RT), the Yi-qi-gu-biao pill_RT improved the forced expiratory volume one-forced vital capacity (FEV_1_/FVC) ratio (mean difference [MD] = 15.343, 95% credible interval [CrI]: 10.233, 20.182). Jin-shui-bao capsule_RT improved tumor necrosis factor-alpha (TNF-α) levels (standard mean difference [SMD] = 2.92, 95% CrI: 2.07, 3.77). Shen-ling-bai-zhu powder_RT improved partial oxygen pressure (MD = 17.17, 95% CrI = 7.43, 26.93). The Yi-fei capsule_RT improved FVC (MD = 0.609, 95% CrI = 0.249, 0.696) and FEV1 (MD = 0.621, 95% CrI = 0.217, 1.023). However, no statistically significant differences were observed between the interventions for the modified Medical Research Council (mMRC) score, peak expiratory flow (PEF), partial pressure of carbon dioxide (PaCO_2_), total effective rate, or reduction in adverse reactions. Based on SUCRA, the Yi-qi-gu-biao pill_RT ranked highest for FEV_1_/FVC (SUCRA = 95.6%) and the mMRC score (SUCRA = 78.6%). The Jin-shui-bao capsule_RT showed advantages in TNF-α levels (SUCRA = 97.4%) and PEF (SUCRA = 69.9%). Shen-ling-bai-zhu powder_RT demonstrated the greatest improvement in PaO_2_ (SUCRA: 99.6%) and PaCO_2_ (SUCRA: 87.1%). Yi-fei capsule_RT was the most effective in improving FVC (SUCRA = 93.2%) and FEV_1_ (SUCRA = 80%). Bu-zhong-yi-qi granule_RT showed the highest SUCRA for improving the total effective rate (82.4%), and bai-ling capsules_RT exhibited the lowest incidence of adverse reactions (72.7%).

**Conclusion:**

Based on the current findings, no specific OPCM has demonstrated noticeable effects across multiple aspects. However, it is evident that OPCM holds considerable potential as an adjunctive treatment for patients with stable COPD. Future high-quality and well-designed RCTs are necessary to further validate our findings.

**Systematic Review Registration:**

https://www.crd.york.ac.uk/PROSPERO/view/CRD42024511142.

## 1 Introduction

Chronic obstructive pulmonary disease (COPD) represents a heterogeneous lung condition marked by chronic respiratory symptoms and irreversible airflow limitation (Bhatt et al., 2023). As reported by the World Health Organization, more than three million deaths worldwide were attributed to COPD in 2019 (Adeloye et al., 2022). COPD mainly affects adults aged 40 and above, especially individuals over 60. It is a progressive condition that primarily poses risks such as respiratory function impairment, continuous decline in lung capacity, a significant reduction in quality of life, and a heightened risk of complications like cardiovascular diseases, respiratory infections, and pulmonary document 4hypertension (Loganathan and Abdul, 2023) Prolonged airflow limitation and hypoxic conditions not only hinder patients’ daily activities but also have a detrimental effect on their mental health, triggering problems like depression and anxiety. Additionally, COPD greatly heightens the risk of mortality, especially during periods of acute exacerbation. Thus, raising awareness and understanding of COPD, taking effective preventive actions, and providing timely diagnosis and treatment are important for reducing the burden of the disease, enhancing patients’ quality of life, and minimizing socio-economic impacts (Rossaki et al., 2021; Rehman et al., 2020).

At present, the treatment interventions for COPD are primarily divided into three main categories: pharmacological treatment, non-pharmacological treatment, and preventive measures. Pharmacological treatment serves as the principal method for managing COPD (Wang et al., 2020). The primary medications utilized are bronchodilators, which include short-acting and long-acting β2 agonists (MacLeod et al., 2021), as well as anticholinergic drugs, effective in easing airway obstruction and improving breathing capacity. Nevertheless, issues such as insufficient patient compliance (O’Toole et al., 2022), high costs of medication (Stolbrink et al., 2022), and prominent side effects (Miravitlles et al., 2021) are commonly encountered in the pharmacological management of COPD. Numerous patients, particularly older adults, struggle to use inhalers properly or adhere to their medication schedules, which affects treatment effectiveness. Non-pharmacological treatments include pulmonary rehabilitation, oxygen therapy, surgical interventions, and breathing exercises, while preventive measures mainly consist of smoking cessation, improving air quality, and occupational protection. Both measures encounter restrictions related to policy resources and patient compliance (Abraham and Symons, 2015), resulting in varied effects for different individuals. Therefore, there is an urgent need to explore treatment options with minimal side effects to alleviate patients’ symptoms and enhance their quality of life.

With the development of traditional Chinese medicine, an increasing number of proprietary Chinese medicines (PCMs) are being widely used to treat various diseases due to minimal side effects, easy accessibility, strong feasibility, high compliance, and low economic burden (Zhang et al., 2022; Yang N. et al., 2021; Gong et al., 2024). For treating stable COPD, Song (2017) has found that Bai-ling capsule (BLC) combined with routine treatment (RT) can effectively inhibit the progression of fibrosis. Huang et al. (2022) suggest that the combined treatment of ambroxol hydrochloride and Bu-fei-huo-xue capsule (BFHXC) can improve lung function, immune function, and sleep quality, demonstrating reliable efficacy. According to Ma et al. (2015), Bu-zhong-yi-qi granule (BZYQG) demonstrates notable clinical efficacy in the treatment of moderate to severe stable COPD. Jin-shui-bao capsule combined with budesonide-formoterol can effectively reduce serum levels of surfactant protein D, hypoxia-inducible factor-1α, and CXC chemokine ligand 12, thereby alleviating inflammatory responses (Liu et al., 2022). Yi-qi-gu-biao pill (YQGBP) can maintain the immune balance of Th17/Treg in the peripheral blood of patients with stable COPD, improving clinical symptoms (Ma and Luo, 2018). Yi-fei capsule (YFC) combined with umeclidinium/vilanterol dry powder inhaler helps regulate serum levels of basic fibroblast growth factor and SIRT1, improving lung function and cellular immune function (Yang et al., 2023). However, there are currently no recommendations for the optimal choice among these effective interventions.

These studies provide valuable evidence regarding the effects of different types of interventions. However, there are no direct comparisons of efficacy and side effects between different interventions. Therefore, conducting a network meta-analysis (NMA) is essential. Through NMA, it is possible to integrate information from both direct and indirect comparisons, thereby digging out the best oral PCM (OPCM) for treating stable COPD.

## 2 Materials and methods

The NMA adhered to the recommendations outlined in the Preferred Reporting Items for Systematic Reviews and Network Meta-Analyses (PRISMA-NMA) (Hutton et al., 2015). This study protocol has been successfully registered with the International Prospective Register of Systematic Reviews (PROSPERO) under the identification code CRD42024511142 (https://www.crd.york.ac.uk/PROSPERO/view/CRD42024511142).

### 2.1 Search strategy

Studies on PCMs for treating stable COPD were retrieved from PubMed, Web of Science, Embase, Cochrane Library, China National Knowledge Infrastructure, Wanfang, and VIP databases from their inception up to 1 April 2024. The retrieval was conducted by combining the search terms with free words, utilizing the following medical subject headings: “Pulmonary Disease,” “Chronic Obstructive,” “Chinese traditional medicine.” The specific search strategy employed can be found in Supplementary Material S1. In addition, to mitigate the risk of omissions, the references of reviews and meta-analyses were cross-checked to ensure that the retrieved studies were as comprehensive as possible.

### 2.2 Inclusion and exclusion criteria

The inclusion and exclusion criteria were established in strict accordance with the PRISMA guidelines, following the PICOS principles. Studies meeting the following criteria would be included: (i) population: patients with COPD; (ii) interventions: An-chuan-zhi-sheng ointment (ACZSO), BLC, BLC_BFHXC, BFHXC, Bu-fei-jian-pi granule_Bu-fei-yi-shen granule_Yi-qi-zi-shen granule (BFJPG_BFYSG_YQZSG), Bu-fei-yi-yang-hua-tan granule (BFYYHTG), BZYQG, Shen-ge-yi-fei capsule (SGYFC), Shen-ling-bai-zhu powder (SLBZP), Fei-kang granule (FKG), Fu-zheng-hua-zhuo ointment (FZHZO), Gu-ben-ke-chuan capsule (GBKCC), Gu-ben-ke-chuan granule (GBKCG), Gu-shen-ding-chuan pill (GSDCP), Ge-jie-ding-chuan capsule (GJDCC), Ke-chuan-ning capsule (KCNC), Jia-wei-shen-ge powder (JWSGP), Jin-kui-shen-qi pill (JKSQP), Jin-shui-bao capsule (JSBC), JSBC_BFHXC, Ping-chuan-yi-qi granule (PCYQG), San-ao tablet (SAT), Su-huang-zhi-ke capsule (SHZKC), Tong-xin-luo capsule (TXLC), Yi-fei ointment (YFO), Yi-fei-huo-xue granule (YFHXG), YFC, YQGBP, Yi-qi-jian-pi granule (YQJPG), Yu-ping-feng granule (YPFG), Zou-fei-ding-chuan ointment (ZFDCO). Supplementary Material S2 provides detailed information for each botanical material, including the complete and valid scientific name, constituent herbs, family and genus, source verification, official pharmacopoeia name, and extract type; (iii) control intervention: RT; (iv) outcome indicators and diagnostic criteria: forced vital capacity (FVC), forced expiratory volume 1 (FEV_1_), FEV_1_/FVC ratio, FEV_1_%, peak expiratory flow (PEF), St George’s Respiratory Questionnaire (SGRQ), the number of acute exacerbations, partial pressure of oxygen (PaO_2_), partial pressure of carbon dioxide (PaCO_2_), interleukin-8 (IL-8), tumor necrosis factor α (TNF-α), COPD Assessment Test (CAT), modified-Medical Research Council (mMRC) score, 6-minute-walk distance (6 MWD), the total effective rate, and adverse reactions; (v) study type: RCTs published in Chinese or English.

The following types of studies would be excluded: (i) animal or cellular experiments, reviews, meta-analyses, guidelines, conference abstracts, letters, responses, opinions, comments, and similar publications; (ii) studies with missing data or significant errors; (iii) studies without a full text; (iv) studies reporting OPCMs fewer than two times; (v) studies with a sample size of 100 cases or fewer; (vi) NRSI (non-randomized studies of interventions).

### 2.3 Study selection and data extraction

Two investigators (HY and JWZ) independently executed the study screening in accordance with the established inclusion and exclusion criteria. The retrieved entries were imported into EndNote X9, where duplicates were removed. The remaining articles underwent a review of the title and abstract to preliminarily exclude those that did not meet the criteria. Subsequently, full texts were searched and reviewed to determine studies eligible for inclusion. During the process of study selection, differing opinions would be resolved through discussion or by seeking the advice of a third investigator (HTY).

Two investigators (HY and JWZ) independently extracted the data from the final included studies, such as first author, publication year, country, disease duration, sample size, gender, age, interventions, control measures, treatment duration, and outcome indicators. If differing opinions arose, they would be resolved through discussion or by seeking the advice of a third investigator (HTY).

### 2.4 Quality assessment

The Cochrane risk of bias tool version 2 (RoB 2.0) (Higgins et al., 2003) was employed to evaluate the included studies from five aspects: bias stemming from the randomization process, bias resulting from deviations from the intended intervention, bias due to missing outcome data, bias in outcome measurement, and bias in the selection of reported results, including any deviation from the registered protocol. For each study, two investigators (HY and JWZ) executed an independent quality assessment, evaluating the aforementioned five aspects and categorizing them as “low risk,” “high risk,” or “potential risk.” For studies with discrepancies, assessments were made after discussion or consultation with a third investigator (HTY), and the results were presented using a risk of bias graph.

### 2.5 Statistical analysis

Utilizing R software (version 4.1.3) with the gemtc package (version 1.0–1) in conjunction with JAGS software, an NMA was conducted based on a Bayesian framework utilizing the Markov Chain Monte Carlo method. Transitivity assessment in an NMA is crucial and substantially influences subsequent analyses (Salanti, 2012). To ensure comparability of different treatments and the validity of indirect conclusions, the transitivity assumption was assessed via detailed comparisons of clinical and methodological characteristics of all eligible studies (including participant attributes and study design) (Caldwell et al., 2005; Jansen and Naci, 2013). A random-effects model was adopted to account for the clinical differences among the eligible studies, including variations in study populations, intervention methods, and assessment approaches. Four Markov chains were employed for the simulation analysis, with an initial value of 2.5 and a refinement iteration step size of 1. A preliminary simulation of 5,000 iterations was conducted for annealing, followed by 20,000 iterations to achieve convergence in the model. Consistency refers to the degree of agreement between direct and indirect evidence. The Deviance Information Criterion (DIC) was used to compare model fit and global consistency (Dempster, 1997). If the DIC difference was less than five points, it was interpreted as fulfilling the consistency criteria, and a consistency model was subsequently employed. In cases where there were closed loops in the network, the node-splitting method was applied to analyze local consistency. A p-value greater than 0.05 indicated no significant inconsistency between direct and indirect evidence. A detailed statistical report, including our R scripts, analysis reports, and the complete output of the inconsistency tests, is provided in Supplementary Material S3.

Binary variables were expressed as risk ratios (RR) along with their corresponding 95% credible intervals (CrI). Continuous variables were reported as weighted mean differences (WMD) or standard mean difference (SMD), accompanied by their respective 95% CrIs. The efficacy of all treatment regimens was simultaneously analyzed using a Bayesian framework-based random-effects model. The analysis results included network plots for each outcome measure, cumulative ranking probability plots, league tables, and comparison-adjusted funnel plots. The surface under the cumulative ranking curve (SUCRA) served as an indicator of cumulative ranking probabilities. Interventions were ranked according to their SUCRA values, and a value closer to 100% indicated a superior intervention. The entire analysis process of this NMA was conducted using Stata 17.0 and R software (R version 4.3.3).

## 3 Results

### 3.1 Study retrieval and screening process

**FIGURE 1:**
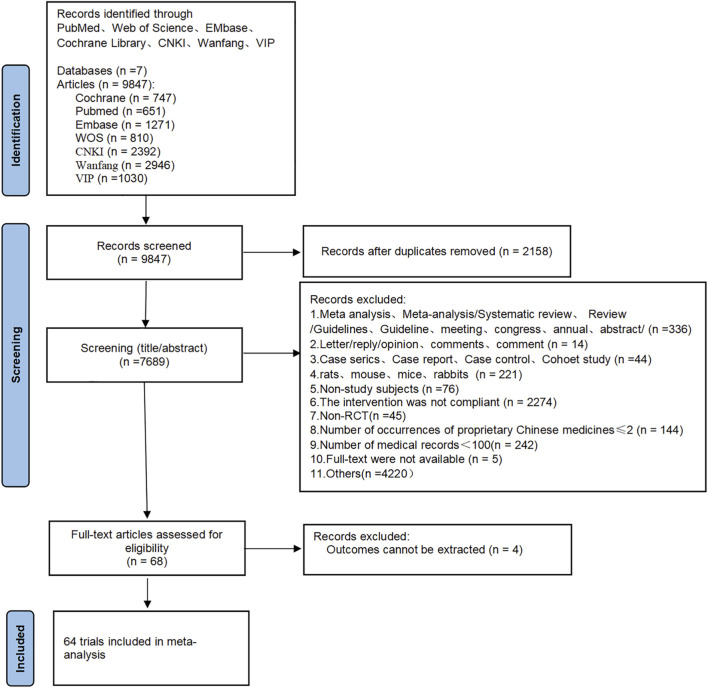
Study screening flowchart.

A total of 9,847 articles were retrieved. After excluding duplicate publications (n = 2,158), 7,689 articles remained. A preliminary review of titles and abstracts excluded 7,621 articles, leaving 68. After conducting a full-text review of the remaining 68 articles, four studies were excluded due to unavailable outcome indicators. Ultimately, 64 articles were included in this analysis. The specific screening process is illustrated in Figure 1.

### 3.2 Basic characteristics of the included studies

The included 64 studies (Song, 2017; Huang et al., 2022; Ma et al., 2015; Liu et al., 2022; Ma and Luo, 2018; Yang et al., 2023; Bai et al., 2016; Liu et al., 2018; Chen et al., 2021; Chen et al., 2019; Chen, 2020; Cheng et al., 2020; Du and Chen, 2015; Fei et al., 2015; Yi et al., 2015; Guan, 2020; Guo et al., 2015; Hao et al., 2016; Peng et al., 2018; Hao et al., 2021; H and Liu, 2012; Huang et al., 2019; Jiang et al., 2017; Huang et al., 2021; Gui et al., 2019; Jia and Zhou, 2022; Ju et al., 2021; Li et al., 2019; Ma and Liu, 2017; Liu, 2014; Liu and Xie, 2015; Luo, 2015; Zhang YL. et al., 2018; Ou et al., 2014; Ou et al., 2015; Qi et al., 2021; Shangguan and Dong, 2015; Song and Xue, 2019; Sun et al., 2014; Wang et al., 2014; Wang et al., 2021; Wang, 2018; Wang J. et al., 2022; Wang MJ. et al., 2022; Wang MH. et al., 2013; Xia et al., 2019; Wang YP. et al., 2013; Wang and Bai, 2019; Zhang et al., 2020; Zhuang et al., 2019; Yang et al., 2018; Xu and Sun, 2015; Yan, 2020; Yang et al., 2013; Yang, 2019; Yang SQ. et al., 2021; Yang, 2021; Ye, 2020; Zhai and Yuan, 2019; Zhang H. et al., 2016; Zhang et al., 2023; Zhang W. et al., 2016; Zhu et al., 2013; Wu and Li, 2018) originated from China and published between 2012 and 2023, encompassing 8,928 patients. The mean age of the participants ranged from 42.34 to 77.50 years, while the duration of illness varied with a mean range of 3.26–21.94 years. BLC is the most commonly used treatment method (Song, 2017; Chen, 2020; Guan, 2020; Hao et al., 2016; Hao et al., 2021; Jia and Zhou, 2022; Luo, 2015; Zhang YL. et al., 2018; Wang et al., 2021; Wang, 2018; Yang, 2019; Yang, 2021; Zhai and Yuan, 2019; Zhang H. et al., 2016), followed by BFHXC (Huang et al., 2022; Chen et al., 2019; Guo et al., 2015; Xia et al., 2019; Wang and Bai, 2019; Yan, 2020; Yang SQ. et al., 2021; Ye, 2020; Zhu et al., 2013), SAT (Shangguan and Dong, 2015; Wang YP. et al., 2013; Zhang W. et al., 2016), SHZKC (Liu et al., 2018; Cheng et al., 2020; Xu and Sun, 2015), JSBC (Liu et al., 2022; Peng et al., 2018; Zhuang et al., 2019), JKSQP (Fei et al., 2015; Zhang et al., 2020), BFYYHTG (Liu, 2014; Wang et al., 2014), FKG (Huang et al., 2019; Qi et al., 2021), ACZSO (Yang et al., 2018), BLC_BFHXC (Bai et al., 2016), BFJPG_BFYSG_YQZSG (Wang MH. et al., 2013), BZYQG (Ma et al., 2015), SGYFC (Huang et al., 2021), SLBZP (Zhang et al., 2023), FZHZO (Du and Chen, 2015), GBKCC (Wang J. et al., 2022), GBKCG (Li et al., 2019), GSDCP (Gui et al., 2019), GJDCC (Song and Xue, 2019), KCNC (Sun et al., 2014), JWSGP (Yi et al., 2015), JSBC_BFHXC (H and Liu, 2012), PCYQG (Liu and Xie, 2015), TXLC (Jiang et al., 2017), YFO (Wang MJ. et al., 2022), YFHXG (Ou et al., 2014; Ou et al., 2015), YFC (Yang et al., 2023), YQGBP (Ma and Luo, 2018; Ma and Liu, 2017), YQJPG (Yang et al., 2013), YPFG (Chen et al., 2021; Wu and Li, 2018), and ZFDCO (Ju et al., 2021). The treatment duration ranged from 2 to 52.14 weeks. The basic characteristic information regarding the included studies is presented in Table 1.

**TABLE 1:** Basic characteristic information regarding the included studies.

First author	Publication year	Country	Disease duration, years (mean ± SD) (E: experimental/observation group) (C: control group)	Sample size	Sex (male/female)	Age Mean ± SD	Treatment		Treatment duration	Main outcomes
							Experimental group	Control group		
Bai SR	2016	China	E: 6.5 ± 3.3 C: 6.3 ± 3.2	E: 90 C: 90	E: 49/41 C: 51/39	E: 58.7 ± 12.5 C: 59.3 ± 13.2	BLC_BFHXC_RT	RT	24 weeks	Efficacy based on TCM syndrome Lung function: FVC, FEV1, and FEV1% Quality of life: SGRQ Number of acute exacerbations of COPD.
Liu XW	2018	China	NM	E: 63 C: 63	96/30	67.12 ± 12.1	SHZKC_RT	RT	12.86 weeks	Lung function: FEV1 and FEV1/FVC Inflammatory factor: IL-8 Dyspnea index: mMRC.
Chen J	2021	China	E: 9.2 ± 2.0 C: 9.6 ± 2.3	E: 137 C: 137	E: 90/47 C: 87/50	E: 62.6 ± 7.5 C: 61.5 ± 7.9	YPFG_RT	RT	12 weeks	Efficacy Lung function: FEV1% and PEF Self-assessment questionnaire: CAT Serum: TNF-α Incidence of adverse reactions: Diarrhea and nausea
Chen QL	2019	China	E: 4.31 ± 1.90 C: 4.47 ± 2.02	E: 55 C: 55	E: 31/24 C: 30/25	E: 62.76 ± 5.33 C: 62.94 ± 5.43	BFHXC_RT	RT	12.86 weeks	Efficacy Lung function: FEV1, PEF, and FEV1/FVC Inflammatory factor: TNF-α Adverse reactions
Chen WH	2020	China	E: 5.95 ± 2.48 C: 5.29 ± 1.25	E: 63 C: 63	E: 30/33 C: 32/31	E: 61.48 ± 5.28 C: 61.26 ± 5.94	BLC_RT	RT	8 weeks	Efficacy Lung function: FVC, FEV1/FVC, and FEV1 Adverse reactions Number of acute exacerbations
Cheng DZ	2020	China	E: 4.48 ± 1.09 C: 4.51 ± 1.14	E: 73 C: 73	E: 46/27 C: 48/25	E: 43.76 ± 6.84 C: 43.81 ± 6.90	SHZKC_RT	RT	12.86 weeks	Efficacy Lung function: FEV1 and FEV1/FVC Quality of life: SGRQ Inflammatory factor: IL-8 and TNF-α Adverse reactions
Du DY	2015	China	E: 3.54 ± 0.46 C: 3.57 ± 0.34	E: 65 C: 65	E: 33/32 C: 31/34	E: 63.58 ± 1.01 C: 63.62 ± 1.02	FZHZO_RT	RT	8 weeks	Efficacy based on TCM syndrome Lung function: FVC, FEV1, and FEV1/FVC Number of acute exacerbations
Fei XF	2015	China	E: 9.21 ± 5.39 C: 9.01 ± 5.50	E: 60 C: 60	E: 45/15 C: 46/14	E: 44.6 ± 5.12 C: 42.34 ± 8.90	JKSQP_RT	RT	52.14 weeks	Lung function: FVC Self-assessment questionnaire: CAT Number of acute exacerbations
Liu W	2022	China	E: 8.79 ± 1.85 C: 9.58 ± 1.90	E: 79 C: 79	E: 47/32 C: 49/30	E: 71.08 ± 4.23 C: 71.59 ± 4.31	JSBC_RT	RT	8 weeks	Efficacy Lung function: FEV1, PEF, and FEV1/FVC 6 MWD Self-assessment questionnaire: CAT Cytokines: TNF-α
Yi X	2015	China	E: 12.56 ± 5.32 C: 13.52 ± 5.86	E: 60 C: 60	E: 35/25 C: 33/27	E: 60.81 ± 8.18 C: 60.41 ± 11.08	JWSGP_RT	RT	12.86 weeks	Lung function: FEV1, FEV1%, PEF, and FEV1/FVC.
Guan FM	2020	China	NM	E: 72 C: 72	E: 41/31 C: 44/28	E: 60.82 ± 9.77 C: 61.92 ± 8.72	BLC_RT	RT	8.57 weeks	Lung function: FEV1, FVC, and FEV1/FVC Efficacy based on TCM syndrome
Guo J	2015	China	NM	E: 60 C: 60	E: 27/33 C: 29/31	E: 61.92 ± 3.27 C: 62.76 ± 5.28	BFHXC_RT	RT	8.57 weeks	Quality of life: SGRQ Efficacy based on TCM syndrome Adverse reactions: nausea, vomiting, and rash
Hao WD	2016	China	E: 11.8 ± 5.8 C: 12.1 ± 6.5	E: 75 C: 75	E: 43/32 C: 40/35	E: 63.1 ± 5.8 C: 61.8 ± 4.0	BLC_RT	RT	8 weeks	Efficacy based on TCM syndrome Lung function: FEV1 pred%, FEV1, and FEV1/FVC Quality of life: SGRQ.
Peng D	2018	China	E: 6 ± 4 C: 5 ± 7	E: 53 C: 53	E: 31/22 C: 32/21	E: 60.1 ± 6.7 C: 59.8 ± 5.8	JSBC_RT	RT	8 weeks	Efficacy based on TCM syndrome Lung function: FEV1, FEV1%, and FEV1/FVC.
Hao Y	2021	China	E: 11.24 ± 3.11 C: 11.42 ± 3.57	E: 55 C: 55	E: 32/23 C: 30/25	E: 71.22 ± 3.76 C: 71.35 ± 3.61	BLC_RT	RT	8.57 weeks	Lung function: FEV1 and FEV1/FVC Incidence of adverse reactions
Hun QG	2012	China	2_	E: 51 C: 50	E: 34/17 C: 34/16	E: 65.41 ± 12.23 C: 64.86 ± 11.84	JSBC_BFHXC_RT	RT	25.71 weeks	Efficacy based on TCM syndrome Lung function: FEV1% and FEV1/FVC BODE index score: MMRC Number of acute exacerbations
Huang HT	2019	China	NM	E: 121 C: 113	E: 75/25 C: 78/22	E: 67.83 ± 7.01 C: 68.10 ± 6.83	FKC_RT	RT	52.14 weeks	Lung function: FVC, FEV1, FEV1% MMRC Number of acute exacerbations Self-assessment questionnaire: CAT Safety indicator: Status of adverse reactions
Jiang MZ	2017	China	E: 5.5 ± 3.2 C: 5.3 ± 3.4	E: 70 C: 70	E: 38/32 C: 40/30	E: 46.0 ± 7.3 C: 45.2 ± 7.6	TXLC_RT	RT	8 weeks	Blood gases: PaCO2 and PaO2 Lung function: FEV1%, FVC, FEV1/FVC Clinical efficacy
Huang XQ	2021	China	E: 12.28 ± 11.18 C: 12.80 ± 10.13	E: 60 C: 60	E: 49/11 C: 47/13	E: 71.22 ± 9.82 C: 69.07 ± 9.99	SGYFC_RT	RT	12.86 weeks	Efficacy based on TCM syndrome Lung function: FEV1% and FEV1/FVC.
Gui K	2019	China	E: 5.17 ± 0.61 C: 5.03 ± 0.58	E: 55 C: 55	E: 32/20 C: 30/21	E: 60.76 ± 6.85 C: 60.44 ± 6.93	GSDCP_RT	RT	12 weeks	Lung function: FEV1% and FEV1/FVC Quality of life: SGRQ Efficacy based on TCM syndrome Serum TNF-α and IL-8
Jia JH	2022	China	E: 5.42 ± 3.45 C: 5.86 ± 3.39	E: 100 C: 100	E: 64/36 C: 65/35	E: 66.35 ± 5.12 C: 64.42 ± 4.98	BLC_RT	RT	12.86 weeks	Observation indicator: PaO2 Lung function: FEV1% and FEV1/FVC Objective scoring and efficacy comparison: CAT, mMRC, and SGRQ Adverse reactions: Mild skin itching, hoarseness of voice, dry throat, and oral ulcers
Jv Y	2021	China	NM	E: 49 C: 58	E: 41/8 C: 47/11	E: 68.43 ± 8.05 C: 69.50 ± 9.47	ZFDCO_RT	RT	52.14 weeks	BODE index score: FEV1%, MMRC, and 6 MWD Inflammatory factor: Serum IL-8 and TNF-α levels
Li L	2019	China	E: 9.87 ± 3.25 C: 9.63 ± 3.10	E: 60 C: 60	E: 38/22 C: 41/19	E: 59.86 ± 5.51 C: 59.17 ± 5.28	GBDCG_RT	RT	12.86 weeks	Clinical efficacy Lung function: FEV1, FEV1/FVC, and PEF Inflammatory factor: TNF-α and IL-8 Adverse reactions
Ma HX	2017	China	E: 9.3 ± 1.8 C: 9.2 ± 1.7	E: 86 C: 86	E: 53/33 C: 51/35	E: 61.5 ± 5.2 C: 61.2 ± 5.4	YQGBP_RT	RT	8 weeks	Lung function: FVC, FEV1, and FEV1/FVC%
Liu SZ	2014	China	E: 12.3 ± 3.5 C: 12.5 ± 3.7	E: 52 C: 52	E: 30/22 C: 29/23	E: 66.3 ± 3.3 C: 65.2 ± 3.5	BFYYHTG_RT	RT	26 weeks	TCM syndrome score Lung function: FVC.
Liu XQ	2015	China	NM	E: 58 C: 58	NM	NM	PCYQG_RT	RT	NM	CAT 6 MWD Lung function: FVC FEV1 and FEV1/FVC Clinical efficacy
Ma HX	2018	China	E: 9.3 ± 1.8 C: 9.2 ± 1.7	E: 86 C: 86	E: 83/3 C: 83/3	E: 61.5 ± 5.2 C: 61.2 ± 5.4	YQGBP_RT	RT	12.86 weeks	Peripheral blood levels of IL-8 and TNF-α Lung function: FVC, FEV1, and FEV1/FVC 6-MWD, mMRC, and CAT
Luo SW	2015	China	E: 14.7 ± 5.3 C: 15.1 ± 5.7	E: 51 C: 51	E: 33/18 C: 34/17	E: 72.3 ± 8.5 C: 73.5 ± 8.9	BLC_RT	RT	8 weeks	Lung function: FEV1 and FEV1/FVC Life indicator: 6-MWD.
Zhang YL	2018	China	E: 4.39 ± 1.72 C: 4.27 ± 1.63	E: 52 C: 52	E: 31/21 C: 29/23	E: 58.13 ± 6.54 C: 57.92 ± 6.31	BLC_RT	RT	12 weeks	Lung function: FVC, FEV1, and FEV1/FVC Clinical efficacy
Ma YF	2015	China	E: 11.6 ± 2.7 C: 11.6 ± 2.7	E: 55 C: 55	E: 36/19 C: 39/16	E: 63.5 ± 15.4 C: 62.7 ± 16.8	BZYQG_RT	RT	12.86 weeks	Efficacy based on TCM syndrome Lung function: FEV, FVC, and FEV1/FVC 6-MWD CAT.
Ou M	2014	China	NM	E: 60 C: 52	E: 37/23 C: 31/21	E: 65.83 ± 9.12 C: 64.95 ± 10.33	YFHXG_RT	RT	26 weeks	Lung function: FEV1, FEV1%, and FEV1/FVC% SGRQ 6 MWT Adverse reactions
Ou M	2015	China	NM	E: 68 C: 60	E: 42/26 C: 37/23	E: 65.80 ± 9.12 C: 64.95 ± 10.33	YFHXG_RT	RT	12 weeks	Lung function: FEV1, FEV1%, and FEV1/FVC% 6 MWT Quality of life: SGRQ Adverse reactions
Qi YL	2021	China	E: 21.49 ± 2.75 C: 21.94 ± 2.69	E: 52 C: 52	E: 25/27 C: 23/29	E: 65.82 ± 2.75 C: 66.05 ± 2.71	FKC_RT	RT	12 weeks	Treatment efficacy Lung function: FEV1, FVC, and FEV1/FVC.
Huang Z	2022	China	E: 3.32 ± 0.93 C: 3.26 ± 0.84	E: 60 C: 60	E: 39/21 C: 41/19	E: 52.16 ± 5.38 C: 52.49 ± 6.37	BFHXC_RT	RT	12.86 weeks	Total effective rate Lung function: FEV1, PEF, FVC Adverse reactions: nausea, vomiting, dyspepsia, and diarrhea
ShangGuan H	2015	China	E: 8.2 ± 3.3 C: 7.2 ± 3.4	E: 102 C: 88	E: 62/38 C: 56/32	E: 63.1 ± 9.1 C: 62.6 ± 8.3	SAT_RT	RT	4.29 weeks	Efficacy Lung function: FEV1, FVC, and FEV1/FVC Blood gases: Pa02 and Pac02 CAT Adverse reactions: Diarrhea, dry mouth, bloating, and poor appetite
Song SL	2017	China	NM	E: 60 C: 60	E: 31/29 C: 32/28	E: 50–73 C: 49–75	BLC_RT	RT	8 weeks	Inflammatory factor: IL-8
Song ZH	2019	China	E: 8.19 ± 2.93 C: 8.29 ± 2.99	E: 60 C: 60	E: 33/27 C: 34/26	E: 67.68 ± 4.75 C: 67.82 ± 4.81	GJDCC_RT	RT	12.86 weeks	Efficacy; Lung function: FVC, FEV1, and FVC/FEV1 6 MWT Adverse reactions: Tremors, palpitations, joint pain, and myalgia
Sun J	2014	China	NM	E: 55 C: 55	E: 34/16 C: 37/14	E: 69.36 ± 8.16 C: 71.55 ± 7.65	KCNC_RT	RT	12.86 weeks	Efficacy BODE index score: FEV1%, MMRC, and 6 MWD CAT.
Wang GA	2014	China	E: 17.3 ± 0.1 C: 16.4 ± 0.3	E: 70 C: 68	E: 40/30 C: 38/30	E: 61.8 ± 1.3 C: 62.6 ± 1.2	BFYYHTG_RT	RT	8.57 weeks	Clinical efficacy Lung function: FEV1, FVC, and FEV1/FVC.
Wang HG	2021	China	E: 9.53 ± 3.58 C: 9.86 ± 3.33	E: 100 C: 100	E: 67/33 C: 65/35	E: 66.55 ± 4.14 C: 67.21 ± 5.48	BLC_RT	RT	8 weeks	Clinical efficacy SGOR Lung function: FEV1 and FVC Adverse reactions: nausea, vomiting, dizziness, constipation, and urinary retention
Wang HT	2018	China	NM	E: 60 C: 60	E: 33/27 C: 34/26	E: 59.31 ± 12.25 C: 60.08 ± 12.36	BLC_RT	RT	12 weeks	Clinical efficacy Lung function: FVC, FEV1,and FEV1/FEV1 Blood gases: PaO2 and PaCO2
Wang J	2018	China	E: 10.83 ± 2.54 C: 10.98 ± 2.49	E: 51 C: 51	E: 28/23 C: 29/22	E: 72.12 ± 3.24 C: 72.71 ± 3.38	GBDCG_RT	RT	12 weeks	Efficacy Lung function: FEV1, PEF, and FEV1/FVC Blood gases: PaO2 and PaCO2 CAT and 6 MWD.
Wang MJ	2022	China	E: 7.1 ± 1.7 C: 6.9 ± 1.9	E: 62 C: 60	E: 42/20 C: 40/20	E: 59.4 ± 7.1 C: 61.3 ± 7.5	YFO_RT	RT	12.86 weeks	Lung function: FEV1, FVC, FEV1%, and FEV1/FVC CAT and SGRQ Adverse reactions
Wang MH	2013	China	E: 13.91 ± 8.83 C: 13.60 ± 10.99	E: 176 C: 176	E: 106/49 C: 116/35	E: 62.74 ± 9.87 C: 64.66.3 ± 8.9	BFJPG_RT BFYSG_RT YQZSG_RT	RT	26 weeks	Number of acute exacerbations Lung function: FEV1, FVC, and FEV1% 6 MWD and MMRC.
Xia WJ	2019	China	E: 8.34 ± 2.72 C: 7.98 ± 3.62	E: 70 C: 70	E: 40/30 C: 42/28	E: 58.42 ± 7.45 C: 56.86 ± 9.79	BFHXC_RT	RT	26 weeks	Clinical efficacy Serum levels of inflammatory cytokines: TNF-α Lung function: PEF, FEV1, and FEV1/FVC Number of acute exacerbations and 6 MWT Adverse reactions: Occurrence of severe adverse reactions, blood routine, liver and kidney function, rash, and mild nausea
Wang YP	2013	China	NM	E: 67 C: 62	91/38	69.4	SAT_RT	RT	8.57 weeks	Lung function: FEV1%, FVC, and FEV1/FVC Blood gases: PaO2 and PaCO2 CAT.
Wang YR	2019	China	NM	E: 60 C: 60	E: 33/27 C: 31/29	E: 63.52 ± 4.38 C: 62.88 ± 5.02	BFHXC_RT	RT	12.86 weeks	Efficacy based on TCM syndrome Lung function: FVC, FEV1, and FEV1% Blood gases: PaO2 and PaCO2
Zhang J	2020	China	E: 7.80 ± 2.25 C: 7.93 ± 2.07	E: 51 C: 51	E: 32/19 C: 30/21	E: 52.16 ± 6.25 C: 52.09 ± 6.38	JKSQP_RT	RT	12.86 weeks	Total effective rate Lung function: FVC, FEV1/FVC, PEF, and FEV1%; mMRC Adverse reactions: Palpitations, headache, hoarseness, discomfort in the epigastric region, and loss of appetite
Zhuang L	2019	China	E: 7.3 ± 2.0 C: 6.8 ± 2.3	E: 60 C: 60	E: 39/21 C: 34/26	E: 58.9 ± 6.5 C: 58.1 ± 6.2	JSBC_RT	RT	12 weeks	Clinical efficacy; mMRC and CAT Lung function: FEV1/FVC, and FEV1% Serum: IL-8 Adverse reactions: Headache and gastrointestinal discomfort
Yang LC	2018	China	E: 13.8 ± 6.1 C: 14.3 ± 5.5	E: 50 C: 50	E: 39/11 C: 35/15	E: 60.1 ± 9.8 C: 58.4 ± 10.3	ACZSO_RT	RT	12.86 weeks	CAT Lung function: FEV1 and FEV1/FVC.
Xu T	2015	China	NM	E: 85 C: 85	92/78	65.7	SHZKC_RT	RT	4.29 weeks	CAT.
Yan QL	2020	China	E: 13.09 ± 3.51 C: 12.14 ± 3.96	E: 58 C: 58	E: 34/24 C: 37/21	E: 66.32 ± 5.48 C: 64.72 ± 5.18	BFHXC_RT	RT	12 weeks	Clinical efficacy Adverse reactions: dry mouth, intestinal obstruction, and constipation
Yang JC	2013	China	E: 14.47 ± 9.79 C: 13.74 ± 10.50	E: 72 C: 72	E: 51/9 C: 47/11	E: 65.68 ± 9.88 C: 65.34 ± 8.73	YQJPG_RT	RT	12.86 weeks	Efficacy based on TCM syndrome Lung function: FEV1, FEV1/FVC, FEV1%, and FVC BODE index score: MMRC and 6 MWD.
Yang L	2023	China	E: 5.67 ± 0.58 C: 5.48 ± 0.62	E: 52 C: 52	E: 40/12 C: 38/14	E: 59.17 ± 5.68 C: 59.94 ± 6.73	YFC_RT	RT	2 weeks	6 MWT and CAT Lung function: FVC and FEV1 Incidence of adverse reactions
Yang S	2019	China	E: 6.08 ± 1.79 C: 6.17 ± 1.83	E: 56 C: 56	E: 32/24 C: 34/22	E: 67.24 ± 5.06 C: 66.53 ± 5.12	BLC_RT	RT	8.57 weeks	Efficacy based on TCM syndrome Lung function: FEV1 and FEV1/FVC Serum IL-8 and TNF-a
Yang SQ	2021	China	E: 5.31 ± 1.17 C: 5.17 ± 1.18	E: 63 C: 63	E: 35/28 C: 34/29	E: 53.14 ± 4.62 C: 53.78 ± 4.39	BFHXC_RT	RT	12 weeks	Clinical total effective rate Lung function: FEV1/FVC, FEV1, and FVC Blood gases: PaO2 and PaCO2 Adverse reactions: nausea, vomiting, and gastrointestinal discomfort
Yang SW	2021	China	E: 8.02 ± 2.31 C: 8.56 ± 1.84	E: 51 C: 51	E: 29/22 C: 26/25	E: 50.01 ± 13.47 C: 49.56 ± 14.26	BLC_RT	RT	26 weeks	Clinical efficacy Lung function: FEV1 and FEV1/FVC 6 MWT Adverse reactions: Headache, palpitations, and tremors
Ye YQ	2020	China	E: 6.6 ± 2.3 C: 6.4 ± 2.1	E: 100 C: 100	E: 52/48 C: 54/46	E: 65.3 ± 8.6 C: 64.6 ± 8.2	BFHXC_RT	RT	12.86 weeks	Clinical efficacy 6 MWD SGRQ.
Zhai XM	2019	China	E: 5.49 ± 1.22 C: 5.53 ± 1.25	E: 62 C: 62	E: 37/25 C: 35/27	E: 71.26 ± 8.14 C: 72.63 ± 7.87	BLC_RT	RT	12 weeks	Clinical efficacy Lung function: FEV1, FEV1/FVC, and FEV1% Incidence of adverse reactions: Palpitations, headachs, tremors, and throat irritation
Zhang H	2016	China	E: 15.5 ± 1.6 C: 15.9 ± 1.9	E: 64 C: 64	E: 40/24 C: 38/26	E: 77.2 ± 3.4 C: 77.5 ± 3.9	BLC_RT	RT	12 weeks	Blood gases: PaO2 and PaCO2 Lung function: FEV1/FVC 6 MWT Number of acute exacerbations Adverse reactions: Gastrointestinal reactions, rash, and dizziness
Zhang LF	2023	China	NM	E: 105 C: 105	E: 64/41 C: 67/38	E: 64.34 ± 4.10 C: 64.18 ± 4.25	SLBZP_RT	RT	12.86 weeks	Efficacy evaluation Blood gases: PaO2 and PaCO2 Lung function: FEV1 and FEV1%
Zhang W	2016	China	E: 7.0 ± 3.1 C: 7.2 ± 3.1	E: 151 C: 128	E: 106/45 C: 79/49	E: 64.6 ± 10.3 C: 63.0 ± 10.0	SAT_RT	RT	2 weeks	Adverse reactions: nausea, mild diarrhea, palpitations, gastrointestinal reactions, palpitations
Zhu DQ	2013	China	E: 9–18 C: 8–19	E: 60 C: 60	E: 32/32 C: 31/29	E: 63 C: 62.5	BFHXC_RT	RT	12.86 weeks	Blood gases: PaO2 and PaCO2 Lung function: FEV1 and FVC 6 MWT Number of acute exacerbations within 1 year
Wu SB	2018	China	E: 13.10 ± 2.75 C: 13.09 ± 2.64	E: 56 C: 56	E: 31/25 C: 32/24	E: 63.92 ± 5.37 C: 63.70 ± 5.26	YPFG_RT	RT	52.14 weeks	Clinical total effective rate Lung function: FEV1/FVC and FEV1%

### 3.3 Assessment results of methodological quality for the included studies

**FIGURE 2:**
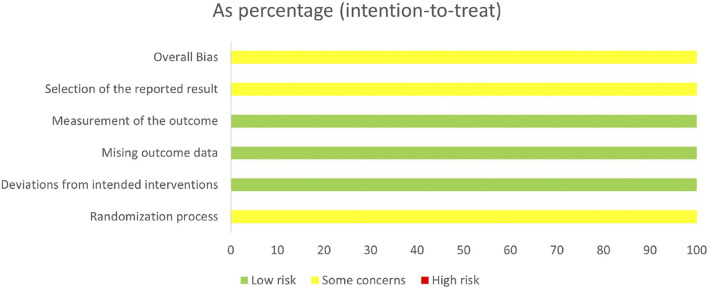
Summary of risk of bias assessment.

The results of the risk of bias assessment for the included 64 studies are available in Figure 2. In terms of bias arising from the randomization process, all studies were assessed as at potential risk due to the absence of random allocation or concealment of group allocation. All studies were unclear regarding the presence of selective reporting, indicating a potential risk of bias in this domain. Regarding bias from deviations from the intended interventions, missing outcome data, and measurement outcomes, all studies were rated as low risk. Overall, the included studies demonstrated a low risk of bias.

### 3.4 NMA results

Before presenting the comprehensive results, the core assumptions of the NMA were evaluated. The transitivity assumption was deemed acceptable since the baseline characteristics of the participants and the key study design features (Table 1) were similar across the trial groups used for indirect comparisons. The DIC was applied to test for global consistency and revealed that the differences in DIC between the consistency and inconsistency models were all less than five for all outcomes. Thus, there was no overall inconsistency between direct and indirect evidence across the networks for all outcomes (Supplementary Material S3). Since the network did not contain closed loops, the node-splitting method was not used to analyze local consistency. In summary, these data are suitable for an NMA.

#### 3.4.1 FVC

**FIGURE 3:**
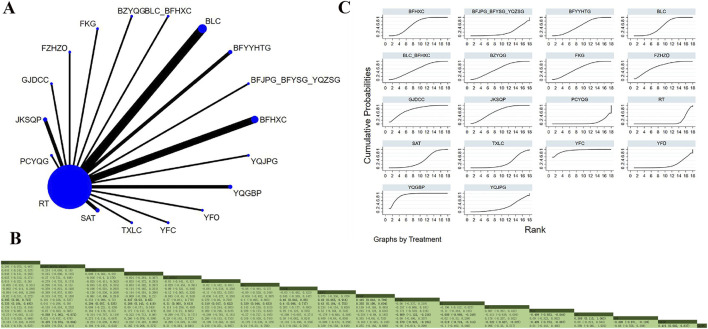
Network plot and network meta-analysis results. **(A)** Network plot for FVC; **(B)** Relative effect of different OPCMs on FVC; **(C)** Cumulative probability line graph.

Twenty-eight studies involving 3,834 participants examined FVC (Ma et al., 2015; Ma and Luo, 2018; Yang et al., 2023; Bai et al., 2016; Chen, 2020; Du and Chen, 2015; Fei et al., 2015; Guan, 2020; Jiang et al., 2017; Ma and Liu, 2017; Liu, 2014; Liu and Xie, 2015; Zhang YL. et al., 2018; Qi et al., 2021; Shangguan and Dong, 2015; Song and Xue, 2019; Wang et al., 2014; Wang et al., 2021; Wang, 2018; Wang MJ. et al., 2022; Wang MH. et al., 2013; Wang YP. et al., 2013; Wang and Bai, 2019; Zhang et al., 2020; Zhuang et al., 2019; Yang et al., 2013; Yang SQ. et al., 2021; Zhu et al., 2013). The analysis indicated low overall heterogeneity (I^2^ = 5%). In addition to RT, 17 OPCMs were included: BLC_BFHXC, BLC, FZHZO, JKSQP, TXLC, YQGBP, BFYYHTG, PCYQG, BZYQG, FKG, BFHXC, SAT, GJDCC, YFC, BFJPG_BFYSG_YQZSG, JKSQP, and YQJPG. The network plot illustrating the various interventions is presented in Figure 3A. The results revealed that YFC demonstrated superior efficacy in improving FVC compared to YFO (MD = 0.589, 95% CrI: 0.115, 1.063), BFJPG_BFYSG_YQZSG (MD = 0.568, 95% CrI: 0.072, 1.063), PCYQG (MD = 0.669, 95% CrI: 0.216, 1.122), RT (MD = 0.609, 95% CrI: 0.249, 0.696), and TXLC (MD = 0.499, 95% CrI: 0.049, 0.953) (Figure 3B). According to SUCRA, YFC (SUCRA: 93.2%), YQGBP (SUCRA: 89.4%), and GJDCC (SUCRAs: 76.7%) were identified as the three most effective interventions for improving FVC (Figure 3C).

#### 3.4.2 FEV_1_

**FIGURE 4:**
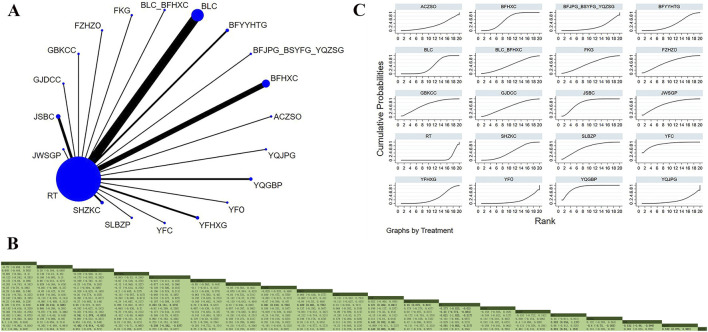
Network plot and network meta-analysis results. **(A)** Network plot for FEV_1_; **(B)** Relative effect of different OPCMs on FEV_1_; **(C)** Cumulative probability line graph.

Thirty-nine studies involving 5,145 participants reported FEV_1_ (Huang et al., 2022; Liu et al., 2022; Ma and Luo, 2018; Yang et al., 2023; Bai et al., 2016; Liu et al., 2018; Chen et al., 2019; Chen, 2020; Cheng et al., 2020; Du and Chen, 2015; Yi et al., 2015; Guan, 2020; Hao et al., 2016; Peng et al., 2018; Hao et al., 2021; Ma and Liu, 2017; Liu, 2014; Luo, 2015; Zhang YL. et al., 2018; Ou et al., 2014; Ou et al., 2015; Qi et al., 2021; Song and Xue, 2019; Wang et al., 2014; Wang et al., 2021; Wang, 2018; Wang J. et al., 2022; Wang MJ. et al., 2022; Wang MH. et al., 2013; Xia et al., 2019; Wang and Bai, 2019; Yang et al., 2018; Yang et al., 2013; Yang, 2019; Yang SQ. et al., 2021; Yang, 2021; Zhai and Yuan, 2019; Zhang et al., 2023; Zhu et al., 2013). The analysis revealed low overall heterogeneity (I^2^ = 0%). In addition to RT, 19 OPCMs were included: BLC_BFHXC, SHZKC, BLC, FZHZO, JSBC, JWSGP, YFHXG, YQGBP, BFYYHTG, GBKCC, FKG, BFHXC, GJDCC, YFO, ACZSO, SLBZP, BFJPG_BFYSG_YQZSG, YFC, and YQJPG. The network plot illustrating the various interventions is presented in Figure 4A. The results implied that, compared to YFO (MD = 0.592, 95% CrI: 0.054, 1.127), YQJPG (MD = 0.591, 95% CrI: 0.03, 1.153), BFJPG_BSYFG_YQZSG (MD = 0.541, 95% CrI: 0.003, 1.076), and RT (MD = 0.621, 95% CrI: 0.217, 1.023), YFC indicated superior efficacy in improving FEV_1_ (Figure 4B). According to SUCRA, YFC (SUCRA: 88.2%), YQGBP (SUCRA: 87.6%), and JSBC (SUCRA: 78%) represented the three most effective interventions for improving FEV_1_ (Figure 4C).

#### 3.4.3 FEV_1_/FVC ratio

**FIGURE 5:**
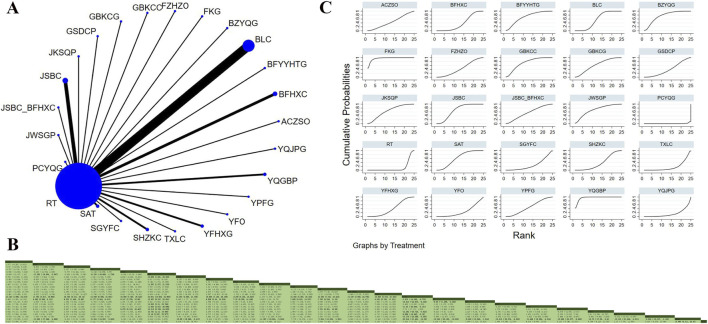
Network plot and network meta-analysis results. **(A)** Network plot for FEV_1_/FVC; **(B)** Relative effect of different OPCMs on FEV_1_/FVC; **(C)** Cumulative probability line graph.

Forty-two studies involving 5,244 participants investigated the FEV_1_/FVC ratio (Ma et al., 2015; Liu et al., 2022; Ma and Luo, 2018; Liu et al., 2018; Chen et al., 2019; Chen, 2020; Cheng et al., 2020; Du and Chen, 2015; Yi et al., 2015; Guan, 2020; Hao et al., 2016; Peng et al., 2018; Hao et al., 2021; H and Liu, 2012; Jiang et al., 2017; Huang et al., 2021; Gui et al., 2019; Jia and Zhou, 2022; Li et al., 2019; Ma and Liu, 2017; Liu and Xie, 2015; Luo, 2015; Zhang YL. et al., 2018; Ou et al., 2014; Ou et al., 2015; Shangguan and Dong, 2015; Wang et al., 2014; Wang J. et al., 2022; Wang MJ. et al., 2022; Xia et al., 2019; Wang YP. et al., 2013; Zhang et al., 2020; Zhuang et al., 2019; Yang et al., 2018; Yang et al., 2013; Yang, 2019; Yang SQ. et al., 2021; Yang, 2021; Zhai and Yuan, 2019; Zhang H. et al., 2016; Wu and Li, 2018). The analysis revealed low overall heterogeneity (I^2^ = 1%). In addition to RT, 24 OPCMs were included: ACZSO, BLC, BFHXC, BFYYHTG, BZYQG, SGYFC, FKG, FZHZO, GBKCC, GBKCG, GSDCP, JWSGP, JKSQP, JSBC, JSBC_BFHXC, PCYQG, SAT, SHZKC, TXLC, YFO, YFHXG, YQGBP, YQJPG, and YPFG. The network plot illustrating the various interventions is presented in Figure 5A. The results indicated that compared to YQJPG (MD = 15.489, 95% CrI: 6.311, 24.47), BFHXC (MD = 11.262, 95% CrI: 4.666, 17.348), BLC (MD = 9.164, 95% CrI: 3.563, 14.507), FZHZO (MD = 10.69, 95% CrI: 2.417, 18.665), GSDCP (MD = 11.387, 95% CrI: 2.818, 19.633), JSBC (MD = 7.554, 95% CrI: 1.291, 13.539), PCYQG (MD = 23.016, 95% CrI: 14.39, 31.401), RT (MD = 15.343, 95% CrI: 10.233, 20.182), SAT (MD = 8.402, 95% CrI: 1.514, 15.045), SGYFC (MD = 13.515, 95% CrI: 4.532, 22.274), SHZKC (MD = 18.484, 95% CrI: 4.314, 18.254), TXLC (MD = 15.299, 95% CrI: 6.999, 23.277), YFHXG (MD = 10.73, 95% CrI: 3.6, 17.56), YFO (MD = 13.133, 95% CrI: 4.511, 21.446), and YPFG (MD = 9.859, 95% CrI: 1.103, 18.337), YQGBP was superior in improving the FEV_1_/FVC ratio (Figure 5B). According to SUCRA, YQGBP (SUCRA: 96.6%), FKG (SUCRA: 95.6%), and BZYQG (SUCRA: 75.7%) might be the three most effective interventions for improving the FEV_1_/FVC ratio (Figure 5C).

#### 3.4.4 PEF

**FIGURE 6:**
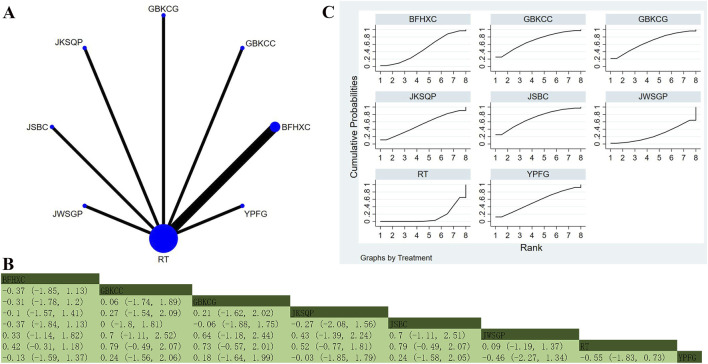
Network plot and network meta-analysis results. **(A)** Network plot for PEF; **(B)** Relative effect of different OPCMs on PEF; **(C)** Cumulative probability line graph.

Nine studies involving 1,206 participants explored PEF (Huang et al., 2022; Liu et al., 2022; Chen et al., 2021; Chen et al., 2019; Yi et al., 2015; Li et al., 2019; Wang J. et al., 2022; Xia et al., 2019; Zhang et al., 2020). The analysis found low overall heterogeneity (I^2^ = 6%). In addition to RT, seven OPCMs were included: BFHXC, GBKCC, GBKCG, JWSGP, JKSQP, JSBC, and YPFG. The network plot illustrating the various interventions is presented in Figure 6A. The results indicated that there were no statistically significant differences among all pairwise interventions (Figure 6B). According to SUCRA, JSBC (SUCRA: 69.9%), GBKCC (SUCRA: 69.8%), and GBKCG (SUCRA: 66.5%) could be the three most effective interventions for improving PEF (Figure 6C).

#### 3.4.5 PaO_2_

**FIGURE 7:**
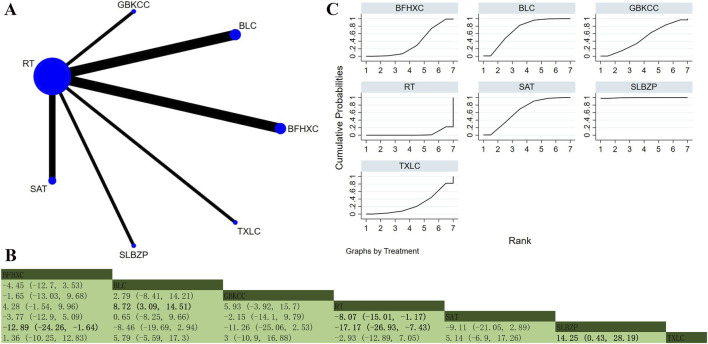
Network plot and network meta-analysis results. **(A)** Network plot for PaO_2_; **(B)** Relative effect of different OPCMs on PaO_2_; **(C)** Cumulative probability line graph.

Eleven studies involving 1,585 participants explored PaO_2_ (Jiang et al., 2017; Jia and Zhou, 2022; Shangguan and Dong, 2015; Wang, 2018; Wang J. et al., 2022; Wang YP. et al., 2013; Wang and Bai, 2019; Yang SQ. et al., 2021; Zhang H. et al., 2016; Zhang et al., 2023; Zhu et al., 2013). The analysis demonstrated low overall heterogeneity (I^2^ = 6%). In addition to RT, six OPCMs were included: BFHXC, BLC, SAT, SLBZP, GBKCC, and TXLC. The network plot illustrating the various interventions is presented in Figure 7A. The results demonstrated that compared to TXLC (MD = 14.25, 95% CrI: 0.43, 28.19), BFHXC (MD = 12.89, 95% CrI: 1.64, 24.26), and RT (MD = 17.17, 95% CrI: 7.43, 26.93), SLBZP exhibited superior efficacy in improving PaO_2_ (Figure 7B). According to SUCRA, SLBZP (SUCRA: 99.6%), BLC (SUCRA: 71%), and SAT (SUCRA: 65.4%) might be the three most effective interventions for improving PaO_2_ (Figure 7C).

#### 3.4.6 PaCO_2_

**FIGURE 8:**
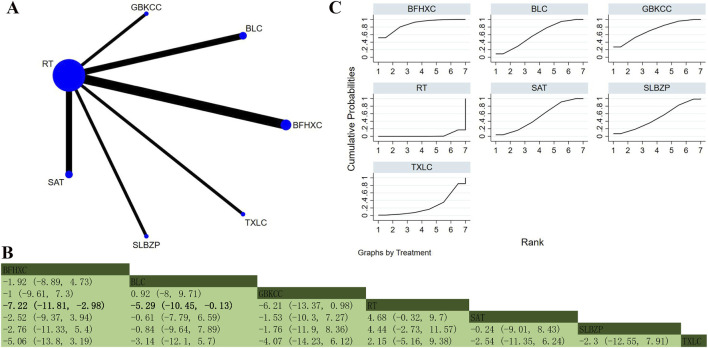
Network plot and network meta-analysis results. **(A)** Network plot for PaCO_2_; **(B)** Relative effect of different OPCMs on PaCO_2_; **(C)** Cumulative probability line graph.

Ten studies involving 1,385 participants reported PaCO_2_ (Jiang et al., 2017; Shangguan and Dong, 2015; Wang, 2018; Wang J. et al., 2022; Wang YP. et al., 2013; Wang and Bai, 2019; Yang SQ. et al., 2021; Zhang H. et al., 2016; Zhang et al., 2023; Zhu et al., 2013). The analysis illustrated low overall heterogeneity (I^2^ = 6%). In addition to RT, six OPCMs were included: BFHXC, BLC, SAT, SLBZP, GBKCC, and TXLC. The network plot illustrating the various interventions is presented in Figure 8A. The results indicated that BFHXC (MD = −7.22, 95% CrI: −11.81, −2.98) and BLC (MD = −5.29, 95% CrI: −10.45, −0.13) demonstrated superior efficacy in improving PaCO_2_ compared to RT, with statistically significant differences noticed (Figure 8B). According to SUCRA, BFHXC (SUCRA: 87.1%), GBKCC (SUCRA: 71.8%), and BLC (SUCRA: 61.1%) may represent the three most effective interventions for improving PaCO_2_ (Figure 8C).

#### 3.4.7 TNF-α

**FIGURE 9:**
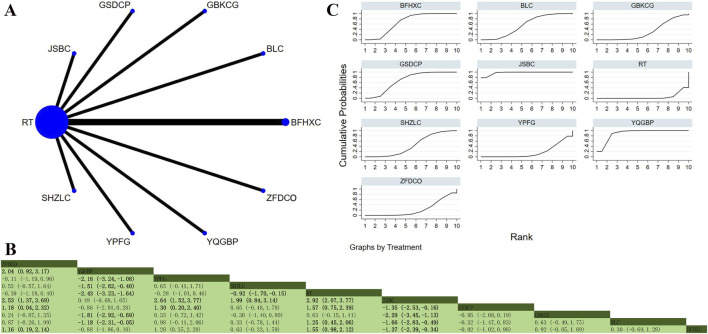
Network plot and network meta-analysis results. **(A)** Network plot for TNF-α; **(B)** Relative effect of different OPCMs on TNF-α; **(C)** Cumulative probability line graph.

Ten studies involving 1,402 participants examined TNF-α (Liu et al., 2022; Ma and Luo, 2018; Chen et al., 2021; Chen et al., 2019; Cheng et al., 2020; Gui et al., 2019; Ju et al., 2021; Li et al., 2019; Xia et al., 2019; Yang, 2019). The analysis revealed low overall heterogeneity (I^2^ = 5%). In addition to RT, nine OPCMs were included: BFHXC, BLC, GBKCG, GSDCP, JSBC, SHZKC, YQGBP, YPFG, and ZFDCO. The network plot illustrating the various interventions is presented in Figure 9A. The results revealed that compared to ZFDCO (SMD = 2.53, 95% CrI: 1.37, 3.68), YPFG (SMD = 2.64, 95% CrI: 1.52, 3.77), SHZLC (SMD = 1.99, 95% CrI: 0.84, 3.14), RT (SMD = 2.92, 95% CrI: 2.07, 3.77), GSDCP (SMD = 1.35, 95% CrI: 0.16, 2.53), GBKCG (SMD = 2.29, 95% CrI: 1.13, 3.45), BLC (SMD = 1.66, 95% CrI: 0.49, 2.83), and BFHXC (SMD = 1.37, 95% CrI: 0.34, 2.39), JSBC exhibited superior efficacy in improving TNF-α levels (Figure 9B). Based on SUCRA, JSBC (SUCRA: 97.4%), YQGBP (SUCRA: 89.7%), and GSDCP (SUCRA: 68%) might be the three most effective interventions for improving TNF-α levels (Figure 9C).

#### 3.4.8 mMRC scores

**FIGURE 10:**
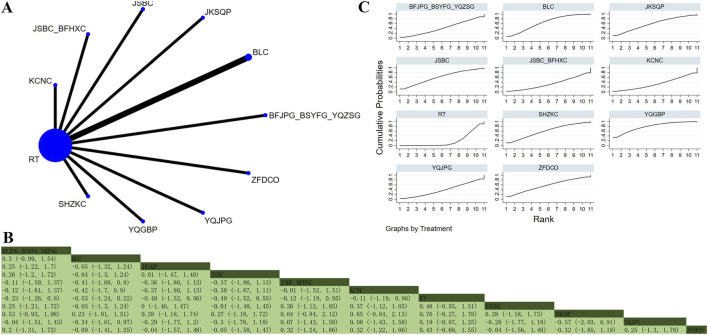
Network plot and network meta-analysis results. **(A)** Network plot for mMRC; **(B)** Relative effect of different OPCMs on mMRC; **(C)** Cumulative probability line graph.

Eleven studies involving 1,573 participants reported mMRC scores (Ma and Luo, 2018; Liu et al., 2018; H and Liu, 2012; Jia and Zhou, 2022; Ju et al., 2021; Sun et al., 2014; Wang, 2018; Wang MH. et al., 2013; Zhang et al., 2020; Zhuang et al., 2019; Yang et al., 2013). The analysis showed low overall heterogeneity (I^2^ = 5%). In addition to RT, ten OPCMs were included: BLC, BFJPG_BFYSG_YQZSG, KCNC, JKSQP, JSBC, JSBC_BFHXC, SHZKC, YQGBP, YQJPG, and ZFDCO. The network plot illustrating the various interventions is presented in Figure 10A. The results indicated that there were no statistically significant differences among all pairwise interventions (Figure 10B). Based on SUCRA, YQGBP (SUCRA: 78.6%), BLC (SUCRA: 64.9%), and JSBC (SUCRA: 61.1%) might be the three most effective interventions for improving mMRC scores (Figure 10C).

#### 3.4.9 Total effective rate

**FIGURE 11:**
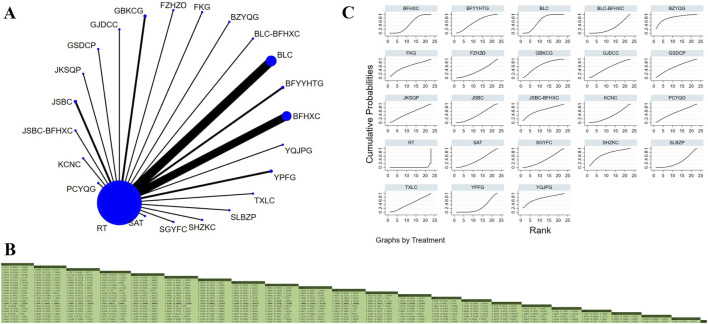
Network plot and network meta-analysis results. **(A)** Network plot for total effective rate; **(B)** Relative effect of different OPCMs on total effective rate; **(C)** Cumulative probability line graph.

Forty-one studies involving 5,375 participants explored this outcome (Huang et al., 2022; Ma et al., 2015; Bai et al., 2016; Chen et al., 2021; Chen et al., 2019; Chen, 2020; Cheng et al., 2020; Du and Chen, 2015; Guan, 2020; Guo et al., 2015; Hao et al., 2016; Peng et al., 2018; H and Liu, 2012; Jiang et al., 2017; Huang et al., 2021; Gui et al., 2019; Li et al., 2019; Liu, 2014; Liu and Xie, 2015; Zhang YL. et al., 2018; Qi et al., 2021; Shangguan and Dong, 2015; Song and Xue, 2019; Sun et al., 2014; Wang et al., 2014; Wang et al., 2021; Wang, 2018; Wang J. et al., 2022; Xia et al., 2019; Wang and Bai, 2019; Zhang et al., 2020; Zhuang et al., 2019; Yan, 2020; Yang et al., 2013; Yang, 2019; Yang SQ. et al., 2021; Yang, 2021; Ye, 2020; Zhai and Yuan, 2019; Zhang et al., 2023; Wu and Li, 2018). The analysis displayed low overall heterogeneity (I^2^ = 0%). In addition to RT, 22 OPCMs were included: BLC, BLC_BFHXC, BFHXC, BFYYHTG, BZYQG, SGYFC, SLBZP, FKG, FZHZO, GBKCG, GSDCP, GJDCC, KCNC, JKSQP, JSBC, JSBC_BFHXC, PCYQG, SAT, SHZKC, TXLC, YQJPG, and YPFG. The network plot illustrating the various interventions is presented in Figure 11A. The findings indicated that BZYQG (MD = 1.30132, 95% CrI: 1.09456, 1.58042), GBKCG (MD = 1.21035, 95% CrI: 1.07922, 1.38312), and JSBC_BFHXC (MD = 1.22109, 95% CrI: 1.00749, 1.51744) effectively enhanced the total effective rate compared to RT, with a statistically significant difference noted (Figure 11B). According to SUCRA, BZYQG (SUCRA: 82.4%), YQJPG (SUCRA: 74%), and SHZKC (SUCRA: 71.9%) could be the three most effective interventions for enhancing the total effective rate (Figure 11C).

#### 3.4.10 Adverse reactions

**FIGURE 12:**
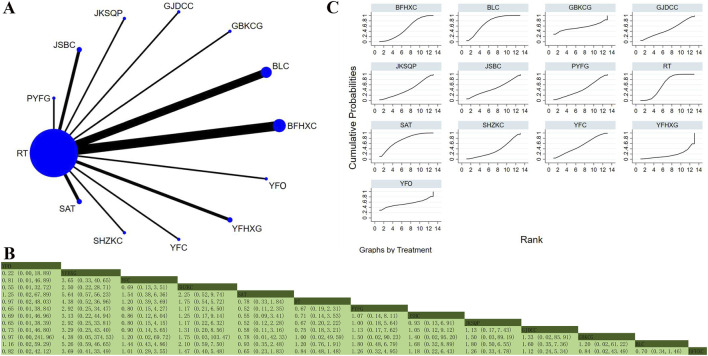
Network plot and network meta-analysis results. **(A)** Network plot for adverse reactions; **(B)** Relative effect of different OPCMs on adverse reactions; **(C)** Cumulative probability line graph.

Twenty-four studies involving 3,400 participants investigated adverse reactions (Huang et al., 2022; Yang et al., 2023; Chen et al., 2021; Chen et al., 2019; Chen, 2020; Cheng et al., 2020; Guo et al., 2015; Hao et al., 2021; Jia and Zhou, 2022; Li et al., 2019; Ou et al., 2014; Ou et al., 2015; Shangguan and Dong, 2015; Song and Xue, 2019; Wang et al., 2021; Wang MJ. et al., 2022; Xia et al., 2019; Zhang et al., 2020; Zhuang et al., 2019; Yan, 2020; Yang SQ. et al., 2021; Yang, 2021; Zhai and Yuan, 2019; Zhang W. et al., 2016). The analysis indicated low overall heterogeneity (I^2^ = 0%). In addition to RT, 12 OPCMs were included: BLC, BFHXC, GBKCG, GJDCC, JKSQP, JSBC, SAT, SHZKC, YFO, YFHXG, YFC, and YPFG. The network plot illustrating the various interventions is presented in Figure 12A. The results indicated that no statistically significant differences were noted among all pairwise interventions (Figure 12B). According to SUCRA, BLC (SUCRA: 72.7%), SAT (SUCRA: 72.6%), and RT (SUCRA: 61.4%) may be the three most effective interventions for reducing the occurrence of adverse reactions (Figure 12C).

#### 3.4.11 Other outcome indicators

The therapeutic effects of various OPCMs on FEV_1_%, SGRQ scores, the number of acute exacerbations, IL-8 levels, CAT, and 6 MWD were also analyzed (Supplementary Material S4).

### 3.5 Publication bias

For assessing publication bias, no evidence of publication bias was found in the adjusted funnel plots (Supplementary Material S5).

## 4 Discussion

According to our knowledge, this is the first NMA comparing the efficacy and safety of different OPCMs as adjunctive therapies for patients with stable COPD. This NMA analyzes the most recent data from 64 RCTs. The findings reveal that, based on RT, YQGBP demonstrates the most effective results in terms of the FEV_1_/FVC ratio or mMRC score. JSBC is identified as the optimal choice for improving TNF-α or PEF. SLBZP is preferred for improving PaO_2_ or PaCO_2_. YFC proves to be the most effective intervention for enhancing FVC or FEV_1_. BFHXC represents the best therapeutic approach for improving FEV_1_% or SGRQ scores. PCYQG is recognized as the optimal selection for improving CAT or 6 MWD assessments. JSBC_BFHXC, GSDCP, and BLC are the optimal choices for reducing the number of acute exacerbations, IL-8 levels, and adverse reactions. BZYQG is most effective in enhancing the total effective rate.

In terms of improving the FEV_1_/FVC ratio, Jiang et al. (2020) demonstrate that YQGBP can enhance airway inflammation levels in patients with COPD, which is consistent with our findings. A related study indicates that airway inflammation can lead to thickening of the airway walls, edema, increased mucus secretion, and bronchial smooth muscle spasms. These changes result in airway narrowing and obstruction, significantly reducing FEV_1_, and consequently leading to a decrease in the FEV_1_/FVC ratio (Lu et al., 2013). Jin et al. (2018) reveal that YQGBP can inhibit the expression of mRNA levels of relevant factors in the JAK/STAT signaling pathway within lung tissue by downregulating IL-23 and IL-17a levels while increasing IFN-γ levels. This modulation leads to an improvement in airway inflammation in COPD patients, thereby enhancing the FEV1/FVC ratio (Jin et al., 2018). The primary metabolites of YQGBP include astragalus root, white atractylodes rhizome, and codonopsis root, which are traditional Chinese medicinal botanical drugs known for their functions in tonifying qi, strengthening the spleen, benefiting the lung, and consolidating the exterior. Wang W. et al. (2018) have conducted a comparative study involving two groups, one receiving astragalus oral solution and the other receiving a placebo. They find that astragaloside IV can effectively improve patients’ FEV_1_ levels by affecting the expression levels of related Treg cells. Additionally, SIRT5 regulates the acetylation status of mitochondrial proteins, improving energy metabolism and autophagy, reducing oxidative stress and inflammation, restoring metabolic balance, and regulating cell apoptosis and proliferation, thereby maintaining the stability of lung tissue. Meng et al. (2024) have suggested that YQGBP improves mitochondrial function in the absence of SIRT5, indirectly alleviates COPD symptoms, and enhances lung function, thereby reducing the mMRC score.

TNF-α plays a key role in the inflammatory response in COPD (Brusselle et al., 2011). In the context of improving TNF-α levels, JSBC_RT demonstrates the most favorable effects. JSBC has the effect of benefiting the lung and kidney, primarily composed of Chinese caterpillar fungus, deer velvet, ginseng, Chinese angelica root, and epimedium leaf. Among these metabolites, Chinese caterpillar fungus is regarded as the sovereign metabolite in this formulation. Chinese caterpillar fungus, as well as its metabolite with other medicinal botanical drugs such as astragalus root and ginseng, has been shown to improve lung function, exercise endurance, quality of life, and symptoms in patients with COPD (Yu et al., 2019; Chen et al., 2014). Metabolites in Chinese caterpillar fungus, such as arachidonic acid, β-sitosterol, and cholesteryl palmitate, exert anti-inflammatory effects in COPD through the PI3K/AKT pathway, p53 signaling pathway, and NF-κB pathway (Wei et al., 2021). By mitigating inflammation, these metabolites reduce airway inflammation, improve airway patency, and enhance PEF.

In terms of improving PaO_2_ and PaCO_2_, SLBZP_RT demonstrates the most effective results. The metabolite of SLBZP includes ginseng, poria, white atractylodes rhizome, dioscorea rhizome, white hyacinth bean, lotus seed, coix seed, amomum fruit, platycodon root, licorice root, and Chinese date. Ginseng, white atractylodes rhizome, and poria serve as the sovereign metabolites, strengthening the spleen and eliminating dampness. Dioscorea rhizome and lotus seed can assist ginseng in boosting qi and supporting the spleen while also stopping diarrhea. White hyacinth bean and coix seed complement white atractylodes rhizome and poria in reinforcing the spleen and alleviating dampness, serving as the minister metabolites. Amomum fruit is used as an adjuvant metabolite to enliven the spleen and harmonize the stomach. As another adjuvant metabolite, platycodon root can ventilate the lung and move qi to regulate the water course and carry medicines upwards, thereby tonifying the lung qi. Licorice root and Chinese date are the courier metabolites to strengthen the spleen, neutralize the middle, and harmonize all kinds of medicines. The metabolite of these botanical drugs is effective in tonifying the spleen and stomach and benefiting the lung qi. The most notable metabolite in this formulation is ginseng. In modern medicine, codonopsis root is often used as a substitute for ginseng. The polysaccharides, flavonoids, and saponins present in codonopsis root exhibit significant antioxidant properties (Zhang X. et al., 2018). Among these metabolites, flavonoid content in codonopsis root is closely linked to its antioxidant activity, indicating that it may serve as an important indicator of the antioxidant capacity of this botanical drug (Wang et al., 2024). Therefore, codonopsis root has the potential to mitigate oxidative stress-induced damage to lung tissue, thereby protecting lung function and subsequently affecting PaO_2_ and PaCO_2_. It also exhibits strong anti-inflammatory effects, primarily by modulating upstream and downstream factors of the NF-κB signaling pathway (Zhang et al., 2024), thereby reducing airway inflammation in COPD patients, improving airway patency, enhancing lung function, increasing PaO_2_, and decreasing PaCO_2_. Codonopsis root is mostly used in the treatment of cardiovascular diseases. It can inhibit apoptosis of H9c2 cardiomyocytes induced by angiotensin II and insulin-like growth factor II (Tsai et al., 2013), enhance myocardial contractility, improve heart function, and increase the oxygen-carrying capacity of the blood.

YFC primarily consists of bitter apricot seed, white mulberry root bark, and fritillaria cirrhosa bulbus. It has the function of clearing away heat and resolving phlegm, tonifying the kidney and the lung, relieving cough, and calming asthma. YFC enhances the antioxidant function of the lung in patients by stimulating pulmonary glutathione peroxidase, thereby increasing the ability to combat inflammatory factors (Ma LH. et al., 2019; Zhu and Zhang, 2018). GSDCP is mainly composed of prepared rehmannia root and prepared aconite accessory root, exhibiting functions such as absorbing and controlling qi and alleviating asthma. It reduces the levels of IL-8 by inhibiting the S100A8/A9 and the NF-κB pathway (Bai et al., 2016; Jin et al., 2018). BFHXC is principally composed of red peony root, astragalus root, and psoralea fruit. It possesses the effects of nourishing the lung, strengthening the kidney, and promoting qi and blood circulation. A pharmacological study has demonstrated that saponins, flavonoid analogs, and polysaccharides present in BFHXC exhibit antibacterial, antioxidant, pulmonary protective, and anti-inflammatory effects (Guo et al., 2015). Furthermore, these metabolites are capable of improving microcirculation and enhancing lung function. The main metabolite of BLC is the fermented powder of Chinese caterpillar fungus, which has the effects of tonifying qi and nourishing yin, replenishing the lung and relieving cough, and strengthening the kidney and replenishing essence. BLC has been indicated to possess anti-inflammatory activity, activating immune cells such as natural killer cells, mononuclear macrophages, and B lymphocytes, thereby modulating the human immune network (Hao et al., 2016). PCYQG primally consists of ephedra and steamed ginseng. It has the effect of promoting the lung and calming asthma, and tonifying the lung and benefiting the qi. Ma JQ. et al. find that PCYQG can reduce airway immune inflammation, alleviate symptoms of dyspnea, improve lung function, and enhance exercise endurance (Ma JQ. et al., 2019). BZYQG is mainly composed of prepared astragalus root and codonopsis root, which possess the function of tonifying the middle and benefiting qi. Ma et al. demonstrate that BZYQG can improve microcirculatory disorders, reduce blood viscosity in COPD patients, lower pulmonary artery pressure, and thereby improve pulmonary ventilation (Ma et al., 2015). JSBC combined with BFHXC can tonify the lung and kidney, have effects such as cough relief and phlegm resolution, anti-inflammatory, antioxidant, bronchodilation of smooth muscle, improve external respiratory function, enhance pulmonary ventilation, and significantly strengthen the body’s humoral immune function (Wang et al., 1995; Yang et al., 2005).

For considerations of CAT or 6 MWD, the combination of PCYQG with conventional Western medicine treatment is preferentially recommended. PCYQG comprises ingredients such as ephedra and red ginseng. Ephedra, with its pungent and warm nature, excels at disseminating lung qi and relieving asthma. It is a key herb for treating wheezing. Red ginseng powerfully tonifies primordial qi, benefits the lungs, strengthens the spleen, and consolidates the foundation, acting as the sovereign drug. Together, these two herbs achieve a synergistic effect. One herb disperses external pathogens without harming vital qi, and the other tonifies lung qi without retaining pathogenic factors. Together, they disseminate lung qi to relieve asthma and tonify lung qi for overall benefit. Modern pharmacological research (Wu et al., 2025) indicates that alkaloids present in ephedra, such as ephedrine and pseudoephedrine, can relax bronchial smooth muscle and alleviate airway spasms, thereby directly ameliorating patients’ dyspnea and reducing CAT scores. Concurrently, components in red ginseng, including saponins and polysaccharides, possess anti-fatigue properties and enhance the body’s tolerance to hypoxia (Lu et al., 2021). This improves patients’ overall physical condition and increases exercise endurance, directly reflected in enhanced 6 MWD.

When considering the frequency of acute exacerbations, JSBC_BFHXC combined with conventional Western medicine treatment may be recommended. The primary ingredient in JSBC is fermented Cordyceps sinensis mycelium. BFHXC consists of red peony root, astragalus, psoralea, and other ingredients. JSBC tonifies the kidneys and lungs, consolidating essence and qi. BFHXC benefits qi, promotes blood circulation, unblocks collaterals, and resolves blood stasis. Together, they tonify the lungs and kidneys, stop coughing, resolve phlegm, and activate blood circulation. From a modern pharmacological perspective, this combined regimen can inhibit the pathological progression of COPD through multiple pathways (Yin et al., 2024; Ren et al., 2024). JSBC can modulate the human immune network and enhance the body’s resistance to disease. BFHXC possesses anti-inflammatory, antioxidant, and microcirculation-improving properties. By enhancing immunity, reducing inflammation, and improving lung function and blood circulation, this combined regimen can significantly reduce the risk of airway infections and acute inflammatory episodes, thereby effectively decreasing the frequency of acute exacerbations.

For airway inflammation mediated by IL-8, a preferential recommendation is given to the co-administration of GSDCP with conventional Western medicine. GSDCP contains traditional Chinese medicines, such as prepared rehmannia root and aconite. Prepared rehmannia root nourishes yin and tonifies the kidneys. Aconite warms and tonifies kidney yang, secures qi, and relieves asthma. Together, these herbs warm the kidneys and secure qi. The core mechanism underlying its anti-inflammatory action is closely related to the regulation of key inflammatory signaling pathways. The NF-κB signaling pathway is a central regulator of the gene expression of pro-inflammatory cytokines like IL-8 (Liu et al., 2017). Endogenous danger signal molecules such as S100A8/A9 can strongly activate the NF-κB pathway by binding to their receptors (e.g., TLR4, RAGE), thereby driving the excessive production and release of IL-8. This recruits neutrophils and exacerbates the inflammatory response (Wang S. et al., 2018). Modern pharmacological research has confirmed that GSDCP and its active components can significantly inhibit the activation of the NF-κB pathway and downregulate the expression of endogenous danger signals like S100A8/A9. By acting on this crucial upstream link, the formulation effectively reduces IL-8 generation, thereby lowering IL-8 levels in the airway and mitigating tissue damage caused by neutrophil infiltration (Bai et al., 2024).

When considering the total effective rate, combining BZYQG with conventional Western medicine is recommended. BZYQG consists of prepared astragalus and codonopsis root, which address the core pathological mechanism of “spleen and stomach qi deficiency with the sinking of clear yang.” In this formula, the prepared astragalus and codonopsis root powerfully tonify the spleen and stomach qi and serve as the sovereign drugs. The aim is to fortify the acquired foundation by strengthening the earth (spleen) to generate metal (lung), thus supplementing the lung qi. This formula can comprehensively improve common systemic symptoms in patients with COPD, such as fatigue, shortness of breath, and loss of appetite, by tonifying the center, benefiting qi, elevating yang, and counteracting sinking. Studies have shown that (Chen et al., 2016) BZYQG can improve microcirculatory disorders, reduce blood viscosity, and alleviate pulmonary hypertension, thereby comprehensively enhancing lung ventilation and overall physical condition. This multi-target improvement of patients’ general function and core pathophysiology contributes to its superior performance in clinical total effective rate.

Regarding adverse reactions, the use of BLC alongside conventional Western medicine may be recommended. The primary ingredient in BLC is fermented Cordyceps sinensis mycelium, which has mild medicinal properties and functions to tonify the lungs and kidneys, as well as benefit essence and qi. Compared to traditional chemical drugs, BLC, as a natural, fermented traditional Chinese medicine preparation, inherently has a lower incidence of adverse reactions. Pharmacological studies indicate that (Tao et al., 2024) BLC primarily exerts its effects by regulating immunity (e.g., activating immune cells, natural killer cells) and exhibiting anti-inflammatory activity, rather than direct cytotoxic effects. Therefore, it causes minimal interference with normal physiological functions and offers good safety, maximizing medication safety while pursuing therapeutic efficacy. Based on the statistical analysis of adverse reactions from 64 articles, 24 articles reported adverse events (Supplementary Material S6). These reactions can be broadly categorized into gastrointestinal and neurological systems. Ranked by frequency of occurrence, the most common adverse reactions were nausea and vomiting, headache, palpitations, throat discomfort, stomach discomfort, and constipation.

Data on the medications used in the observation groups were compiled from the 64 included studies. It was found that varied baseline treatments were employed. In the control groups, 11 studies did not specify the medications used. Nine studies utilized budesonide/formoterol, seven used tiotropium bromide, and seven employed salmeterol/fluticasone. An additional 34 articles utilized 26 different classes of drugs. These varying medications could introduce heterogeneity. Nevertheless, the results of this investigation indicate that adding PCM to baseline treatment consistently yielded improvement. The differences in Western medicine regimens across groups actually reflect the diversity of the included populations. For instance, within comparative experiments evaluating the addition of BLC, differences in sex, age (50–76 years), and disease duration (1–10 years) were noted in Wenhui Chen et al.’s study (Chen, 2020), in which the baseline treatment was tiotropium bromide nebulizer. Fumin Guan et al.’s study (Guan, 2020) included patients ranging in age from 44 to 73 years old. The Western medical treatment included budesonide inhaler, with the co-administration of salbutamol or theophylline derivatives as necessary. Wendong Hao et al.’s research (Hao et al., 2016) included 150 patients with an average age of approximately 62 years and an average disease duration of 12.6 years. Their baseline Western medicine regimen was budesonide/formoterol dry powder inhaler. Due to the older age and relatively shorter disease duration of the patients included in Chen (2020)’s study, which suggests a potentially more stable condition, tiotropium bromide monotherapy was chosen as the baseline treatment instead of a more potent combination therapy. Tiotropium bromide has fewer side effects and is suitable for patients in the stable phase. In the studies by Guan (2020) and Hao et al. (2016), more potent combination inhalers like budesonide/formoterol were selected for patients with more complex conditions and a risk of acute exacerbation, with the addition of bronchodilators like salbutamol as needed. Similarly, in comparative experiments involving the addition of BFHXC, the studies by Chen et al. (2019), Guo et al. (2015), and Huang et al. (2022) all adopted the authoritative “Guidelines for the Diagnosis and Treatment of Chronic Obstructive Pulmonary Disease” as their diagnostic basis. These studies primarily included patients in the stable phase of COPD. This ensured comparability in disease stage and severity. Regarding exclusion criteria, all three studies commonly excluded patients with severe cardiovascular, cerebrovascular, liver, or kidney diseases, other lung diseases (e.g., lung cancer, tuberculosis), pregnant or lactating women, and individuals with psychiatric or cognitive impairments that prevented cooperation. These criteria effectively filtered out complex factors that could influence baseline levels, making the study populations more homogeneous in terms of disease status and overall health. Although individual studies had specific exclusion criteria (e.g., anticoagulant medication use), these did not systematically alter the baseline characteristics of the populations and thus did not affect their overall comparability. Therefore, the studies by these three authors were largely consistent in terms of the disease severity and accompanying symptoms of their populations. These variations suggest that the choice of baseline medications in different studies is likely closely related to factors such as the age and disease duration of the included individuals. Nevertheless, PCMs consistently demonstrated significant therapeutic effects in combination therapy, suggesting that their therapeutic efficacy is minimally influenced by the heterogeneity of baseline Western medications. This indirectly suggests that future researchers can select different Western medications based on individual patient circumstances without affecting the therapeutic outcomes of adding PCMs.

### 4.1 Strengths and limitations

This article represents the first NMA evaluating the efficacy and safety of OPCM as an adjunctive treatment for patients with stable COPD. The optimal intervention methods for improving FVC, FEV_1_, the FEV_1_/FVC ratio, FEV_1_%, PEF, SGRQ scores, the number of acute exacerbations, PaO_2_, PaCO_2_, IL-8 levels, TNF-α levels, CAT scores, mMRC scores, 6 MWD, the total effective rate, and adverse reactions have been identified. However, this NMA still has some limitations. Firstly, in the included studies, the number of studies focusing on indicators such as PaO_2_, PaCO_2_, IL-8 levels, PEF, SGRQ scores, and the number of acute exacerbations was relatively small, which may certainly impact our conclusions. Secondly, although all the included studies are RCTs, some of the articles did not implement blinding, which could lead to potential bias. Thirdly, although subgroup analysis is considered to explore differences based on factors such as gender, age, region, race, and study design, the limitations in the reported data of the included studies prevent detailed analysis. Fourthly, the limited number of articles regarding adverse reactions did not support conducting subgroup analyses. Therefore, they are reported only in tabular form (Supplementary Material S6). The severity of adverse reactions was also not reported in the original studies, precluding further discussion. Fifthly, variations in measurement time points were observed across studies concerning follow-up duration: only four papers mentioned multiple time points, while the remaining literature defined only two time points (i.e., pre-treatment and post-treatment). Finally, this study exclusively selected articles published in Chinese or English. The included studies were all conducted in China, which may have introduced regional bias and selection bias.

## 5 Conclusion

Our results indicate that YQGBP_RT is the preferred option for improving the FEV_1_/FVC ratio or mMRC scores. JSBC_RT is the first choice for improving TNF-α levels or PEF. SLBZP_RT is favored for improving PaO_2_ or PaCO_2_. YFC_RT is the favored option for enhancing FVC or FEV_1_. BFHXC_RT treatment is preferentially recommended when FEV_1_% or SGRQ is considered. For the improvement of CAT or 6 MWD, PCYQG_RT is the preferred option. JSBC_BFHXC_RT is recommended as the preferred option to reduce the number of acute exacerbations. In terms of reducing IL-8, GSDCP_RT is the preferred option. To improve the total effective rate, BZYQG-RT is recommended as a priority. BLC_RT is the preferred option to reduce adverse reactions. However, due to the influence of both the quantity and quality of existing studies, more high-quality, large-scale double-blind RCTs are required to provide further evidence. This study is based entirely on the Chinese region, population, and medical context, so its applicability to other regions is unclear. We suggest that researchers from other countries conduct related research in the future. Based on our findings, we recommend the oral traditional Chinese medicine with the highest cumulative probability ranking. However, due to the lack of statistically significant differences in the current results, future studies require further discussion. Additionally, future analyses and recommendations should be considered in policy.

## Data Availability

The original contributions presented in the study are included in the article/Supplementary Material, further inquiries can be directed to the corresponding author.
