# Supplementary material for: Efficacy and safety of oral proprietary Chinese medicines in the treatment of stable chronic obstructive pulmonary disease: a network meta-analysis
Source: Front Pharmacol. 2026 Jan 21;16:1690739. doi: 10.3389/fphar.2025.1690739 (PMC12868190; doi:10.3389/fphar.2025.1690739)
Supplement: Supplementary file 1 [file Table1.docx]

**Supplementary Document 1** Search strategies

**Pubmed**

**#1** "Pulmonary Disease, Chronic Obstructive"[Mesh]

**#2** "Pulmonary Disease, Chronic Obstructive"OR"Chronic Obstructive Pulmonary disease*"OR"Chronic Obstructive Lung disease*"OR "chronic obstructive respiratory disease*"OR"Pulmonary Emphysema"OR"Chronic bronchitis"OR"Chronic Obstructive Airway"OR "COPD"[Title/Abstract]

**#3** #1 OR #2

**#4** "Chinese traditional medicine"[Mesh]

**#5**"Chinese traditional medicine"OR"Chinese medicine"OR"Chinese patent medicine"OR "Proprietary Chinese medicine"OR"Chinese patent drug"OR"Chinese herbal"OR"capsule"OR "granule"OR "pills"[Title/Abstract]

**#6** #4 OR #5

**#7** #3AND #6

**Cochrane Library**

**#1** MeSH descriptor: Pulmonary Disease, Chronic Obstructive

**#2** "Pulmonary Disease, Chronic Obstructive"OR"Chronic Obstructive Pulmonary disease*"OR"Chronic Obstructive Lung disease*"OR "chronic obstructive respiratory disease*"OR"Pulmonary Emphysema"OR"Chronic bronchitis"OR"Chronic Obstructive Airway"OR "COPD" :ti,ab,kw

**#3** #1 OR #2

**#4** MeSH descriptor: Chinese traditional medicine

**#5** "Chinese traditional medicine"OR"Chinese medicine"OR"Chinese patent medicine"OR "Proprietary Chinese medicine"OR"Chinese patent drug"OR"Chinese herbal"OR"capsule"OR "granule"OR "pills":ti,ab,kw

**#6** #4 OR #5

**#7** #3 AND #6

**Web of Science**

( "Pulmonary Disease, Chronic Obstructive"OR"Chronic Obstructive Pulmonary disease*"OR"Chronic Obstructive Lung disease*"OR "chronic obstructive respiratory disease*"OR"Pulmonary Emphysema"OR"Chronic bronchitis"OR"Chronic Obstructive Airway"OR "COPD" ) AND ("Chinese traditional medicine"OR"Chinese medicine"OR"Chinese patent medicine"OR "Proprietary Chinese medicine"OR"Chinese patent drug"OR"Chinese herbal"OR"capsule"OR "granule"OR "pills")

**Embase**

#1 'Pulmonary Disease, Chronic Obstructive':exp

#2 'Pulmonary Disease, Chronic Obstructive' OR 'Chronic Obstructive Pulmonary disease*'OR 'Chronic Obstructive Lung disease*'OR 'chronic obstructive respiratory disease*'OR 'Pulmonary Emphysema' OR 'Chronic bronchitis' OR 'Chronic Obstructive Airway' OR 'COPD':ab,ti

#3 #1 OR #2

#4 'Chinese traditional medicine':exp

#5'Chinese traditional medicine 'OR' Chinese medicine' OR 'Chinese patent medicine' OR 'Proprietary Chinese medicine' OR 'Chinese patent drug' OR 'Chinese herbal' OR 'capsule' OR 'granule' OR 'pills':ab,ti

#6 #4 OR #5

#7 #3AND #6

**VIP**

（慢性阻塞性肺疾病 OR 慢阻肺 OR COPD）AND（中医 OR 中药 OR 中成药 颗粒 OR 胶囊 OR 片 OR 丸 OR 散 OR 口服液 OR 膏 OR 合剂）AND（稳定期 OR 稳定阶段 OR 稳定时期 OR 平稳期）

**CNKI**

（慢性阻塞性肺疾病 + 慢阻肺 + COPD）AND（中医 + 中药 + 中成药 + 颗粒 + 胶囊 + 片 + 丸 + 散 + 口服液 + 膏 + 合剂）AND（稳定期 + 稳定阶段 + 稳定时期 + 平稳期）

**Wanfang**

（慢性阻塞性肺疾病 OR 慢阻肺 OR COPD）AND（中医 OR 中药 OR 中成药 颗粒 OR 胶囊 OR 片 OR 丸 OR 散 OR 口服液 OR 膏 OR 合剂）AND（稳定期 OR 稳定阶段 OR 稳定时期 OR 平稳期）
