# Supplementary material for: Efficacy and safety of oral proprietary Chinese medicines in the treatment of stable chronic obstructive pulmonary disease: a network meta-analysis
Source: Front Pharmacol. 2026 Jan 21;16:1690739. doi: 10.3389/fphar.2025.1690739 (PMC12868190; doi:10.3389/fphar.2025.1690739)
Supplement: Supplementary file 3 [file Table2.docx]

**Supplementary Document 2** Botanical Drugs Included Preparation Methods of Herbal Formulas, Plant Medicine Ingredients, and Classification and Usage of Plant Parts

| Chinese Name | Latin name | Family and parts used in medicine. |
| --- | --- | --- |
| 太子参 | Pseudostellaria heterophylla (Miq.) Pax | Pseudostellaria heterophylla (Miq.) Pax [Caryophyllaceae; Pseudostellariae radix] |
| 地黄 | Rehmannia glutinosa Libosch. | Rehmannia glutinosa Libosch. [Orobanchaceae; Rehmanniae radix] |
| 麦冬 | Ophiopogon japonicus (L. f.) Ker Gawl. | Ophiopogon japonicus (L. f.) Ker Gawl. [Asparagaceae; Ophiopogonis radix] |
| 山萸肉 | Cornus officinalis Siebold & Zucc. | Cornus officinalis Siebold & Zucc. [Cornaceae; Corni fructus] |
| 牛膝 | Achyranthes bidentata Blume | Achyranthes bidentata Blume [Amaranthaceae; Achyranthis bidentatae radix] |
| 枸杞子 | Lycium barbarum L. | Lycium barbarum L. [Solanaceae; Lycii fructus] |
| 五味子 | Schisandra chinensis (Turcz.) Baill. | Schisandra chinensis (Turcz.) Baill. [Schisandraceae; Schisandrae chinensis fructus] |
| 胡桃仁 | Juglans regia L. | Juglans regia L. [Juglandaceae; Juglandis semen] |
| 当归 | Angelica sinensis (Oliv.) Diels | Angelica sinensis (Oliv.) Diels [Apiaceae; Angelicae sinensis radix] |
| 磁石 | Magnetitum | Magnetitum [Mineralia; Magnetitum] |
| 紫菀 | Aster tataricus L. f. | Aster tataricus L. f. [Asteraceae; Asteris radix et rhizoma] |
| 款冬花 | Tussilago farfara L. | Tussilago farfara L. [Asteraceae; Farfarae flos] |
| 地龙 | Pheretima aspergillum (E. Perrier) | Pheretima aspergillum (E. Perrier) [Megascolecidae; Pheretima] |
| 沉香 | Aquilaria sinensis (Lour.) Spreng. | Aquilaria sinensis (Lour.) Spreng. [Thymelaeaceae; Aquilariae lignum resinatum] |
| 阿胶 | Equus asinus L. | Asini corii colla [Equidae; Asini corii colla] |
| 鹿角胶 | Cervus nippon Temminck | Cervi cornu colla [Cervidae; Cervi cornu colla] |
| 冬虫夏草 | Ophiocordyceps sinensis (Berk.) G.H. Sung, J.M. Sung, Hywel-Jones & Spatafora | Ophiocordyceps sinensis (Berk.) G.H. Sung, J.M. Sung, Hywel-Jones & Spatafora [Ophiocordycipitaceae; Cordyceps] |
| 黄芪 | Astragalus membranaceus (Fisch.) Bunge | Astragalus membranaceus (Fisch.) Bunge [Fabaceae; Astragali Radix] |
| 补骨脂 | Psoralea corylifolia L. | Psoralea corylifolia L. [Fabaceae; Psoraleae Fructus] |
| 党参 | Codonopsis pilosula (Franch.) Nannf. | Codonopsis pilosula (Franch.) Nannf. [Campanulaceae; Codonopsis Radix] |
| 白术 | Atractylodes macrocephala Koidz. | Atractylodes macrocephala Koidz. [Asteraceae; Atractylodis Macrocephalae Rhizoma] |
| 茯苓 | Wolfiporia cocos (F.A. Wolf) Ryvarden & Gilb. | Wolfiporia cocos (F.A. Wolf) Ryvarden & Gilb. [Polyporaceae; Poria] |
| 川贝 | Fritillaria cirrhosa D. Don | Fritillaria cirrhosa D. Don [Liliaceae; Fritillariae Cirrhosae Bulbus] |
| 人参 | Panax ginseng C.A.Mey. | Panax ginseng C.A.Mey. [Araliaceae; Ginseng Radix] |
| 黄精 | Polygonatum sibiricum Red. | Polygonatum sibiricum Red. [Asparagaceae; Polygonati Rhizoma] |
| 巴戟天 | Morinda officinalis How | Morinda officinalis How [Rubiaceae; Morindae Radix] |
| 枇杷叶 | Eriobotrya japonica (Thunb.) Lindl. | Eriobotrya japonica (Thunb.) Lindl. [Rosaceae; Eriobotryae Folium] |
| 葶苈子 | Descurainia sophia (L.) Webb ex Prantl | Descurainia sophia (L.) Webb ex Prantl [Brassicaceae; Lepidii Semen / Descurainiae Semen] |
| 桔梗 | Platycodon grandiflorus (Jacq.) A. DC. | Platycodon grandiflorus (Jacq.) A. DC. [Campanulaceae; Platycodonis Radix] |
| 杜仲 | Eucommia ulmoides Oliv. | Eucommia ulmoides Oliv. [Eucommiaceae; Eucommiae Cortex] |
| 甘草 | Glycyrrhiza uralensis Fisch. | Glycyrrhiza uralensis Fisch. [Fabaceae; Glycyrrhizae Radix et Rhizoma] |
| 大枣 | Ziziphus jujuba Mill. | Ziziphus jujuba Mill. [Rhamnaceae; Jujubae Fructus] |
| 升麻 | Cimicifuga foetida L. | Cimicifuga foetida L. [Ranunculaceae; Cimicifugae Rhizoma] |
| 柴胡 | Bupleurum chinense DC. | Bupleurum chinense DC. [Apiaceae; Bupleuri Radix] |
| 陈皮 | Citrus × aurantium L. 'Chenpi' | Citrus × aurantium L. 'Chenpi' [Rutaceae; Citri Reticulatae Pericarpium] |
| 生姜 | Zingiber officinale Roscoe | Zingiber officinale Roscoe [Zingiberaceae; Zingiberis Rhizoma Recens] |
| 红参 | Panax ginseng C.A.Mey. | Panax ginseng C.A.Mey. [Araliaceae; Ginseng Radix et Rhizoma Rubra] |
| 蛤蚧 | Gekko gecko Linnaeus | Gekko gecko Linnaeus [Gekkonidae; Gecko] |
| 三七 | Panax notoginseng (Burkill) F.H.Chen | Panax notoginseng (Burkill) F.H.Chen [Araliaceae; Notoginseng Radix et Rhizoma] |
| 川芎 | Ligusticum chuanxiong Hort. | Ligusticum chuanxiong Hort. [Apiaceae; Chuanxiong Rhizoma] |
| 前胡 | Peucedanum praeruptorum Dunn | Peucedanum praeruptorum Dunn [Apiaceae; Peucedani Radix] |
| 砂仁 | Amomum villosum Lour. | Amomum villosum Lour. [Zingiberaceae; Amomi Fructus] |
| 山药 | Dioscorea opposita Thunb. | Dioscorea opposita Thunb. [Dioscoreaceae; Dioscoreae Rhizoma] |
| 薏苡仁 | Coix lacryma-jobi L. | Coix lacryma-jobi L. [Poaceae; Coicis Semen] |
| 西洋参 | Panax quinquefolius L. | Panax quinquefolius L. [Araliaceae; Panacis Quinquefolii Radix] |
| 灵芝 | Ganoderma lucidum (Leyss. ex Fr.) Karst. | Ganoderma lucidum (Leyss. ex Fr.) Karst. [Ganodermataceae; Ganoderma] |
| 丹参 | Salvia miltiorrhiza Bunge | Salvia miltiorrhiza Bunge [Lamiaceae; Salviae Miltiorrhizae Radix et Rhizoma] |
| 半夏 | Pinellia ternata (Thunb.) Breit. | Pinellia ternata (Thunb.) Breit. [Araceae; Pinelliae Rhizoma] |
| 五指毛桃 | Ficus hirta Vahl | Ficus hirta Vahl [Moraceae; Fici Hirtae Radix] |
| 杏仁 | Prunus armeniaca L. | Prunus armeniaca L. [Rosaceae; Armeniacae Semen Amarum] |
| 龟板胶 | Chinemys reevesii (Gray) | Chinemys reevesii (Gray) [Geoemydidae; Testudinis Carapax et Plastrum Colla] |
| 黑顺片 | Aconitum carmichaelii Debx. | Aconitum carmichaelii Debx. [Ranunculaceae; Aconiti Lateralis Radix Praeparata] |
| 车前子 | Plantago asiatica L. | Plantago asiatica L. [Plantaginaceae; Plantaginis Semen] |
| 益智仁 | Alpinia oxyphylla Miq. | Alpinia oxyphylla Miq. [Zingiberaceae; Alpiniae Oxyphyllae Fructus] |
| 肉桂 | Cinnamomum cassia (L.) J. Presl | Cinnamomum cassia (L.) J. Presl [Lauraceae; Cinnamomi Cortex] |
| 泽泻 | Alisma orientale (Sam.) Juz. | Alisma orientale (Sam.) Juz. [Alismataceae; Alismatis Rhizoma] |
| 金樱子 | Rosa laevigata Michx. | Rosa laevigata Michx. [Rosaceae; Rosae Laevigatae Fructus] |
| 瓜蒌子 | Trichosanthes kirilowii Maxim. | Trichosanthes kirilowii Maxim. [Cucurbitaceae; Trichosanthis Semen] |
| 麻黄 | Ephedra sinica Stapf | Ephedra sinica Stapf [Ephedraceae; Ephedrae Herba] |
| 烈香杜鹃 | Rhododendron anthopogonoides Maxim. | Rhododendron anthopogonoides Maxim. [Ericaceae; Rhododendri Anthopogonoidis Folium] |
| 黄芩 | Scutellaria baicalensis Georgi | Scutellaria baicalensis Georgi [Lamiaceae; Scutellariae Radix] |
| 浙贝母 | Fritillaria thunbergii Miq. | Fritillaria thunbergii Miq. [Liliaceae; Fritillariae Thunbergii Bulbus] |
| 百部 | Stemona sessilifolia (Miq.) Miq. | Stemona sessilifolia (Miq.) Miq. [Stemonaceae; Stemonae Radix] |
| 莪术 | Curcuma phaeocaulis Val. | Curcuma phaeocaulis Val. [Zingiberaceae; Curcumae Rhizoma] |
| 淫羊藿 | Epimedium brevicornu Maxim. | Epimedium brevicornu Maxim. [Berberidaceae; Epimedii Herba] |
| 吉祥草 | Reineckea carnea (Andrews) Kunth | Reineckea carnea (Andrews) Kunth [Asparagaceae; Reineckeae Herba] |
| 紫河车 | Homo sapiens L. | Homo sapiens L. [Hominidae; Hominis Placenta] |
| 附子 | Aconitum carmichaelii Debx. | Aconitum carmichaelii Debx. [Ranunculaceae; Aconiti Lateralis Radix Praeparata] |
| 桂枝 | Cinnamomum cassia (L.) J. Presl | Cinnamomum cassia (L.) J.Presl [Lauraceae; Cinnamomi Ramulus] |
| 穿山龙 | Dioscorea nipponica Makino | Dioscorea nipponica Makino [Dioscoreaceae; Dioscoreae Nipponicae Rhizoma] |
| 厚朴 | Magnolia officinalis Rehder & E.H.Wilson | Magnolia officinalis Rehder & E.H.Wilson [Magnoliaceae; Magnoliae Officinalis Cortex] |
| 紫苏叶 | Perilla frutescens (L.) Britton | Perilla frutescens (L.) Britton [Lamiaceae; Perillae Folium] |
| 苏子 | Perilla frutescens (L.) Britton | Perilla frutescens (L.) Britton [Lamiaceae; Perillae Fructus] |
| 水蛭 | Homo sapiens L. | Hirudo nipponica Whitman [Hirudinidae; Hirudo] |
| 全蝎 | Buthus martensii Karsch | Buthus martensii Karsch [Buthidae; Scorpio] |
| 赤芍 | Paeonia lactiflora Pall. | Paeonia lactiflora Pall. [Paeoniaceae; Paeoniae Radix Rubra] |
| 蝉蜕 | Cryptotympana pustulata Fabricius | Cryptotympana pustulata Fabricius [Cicadidae; Cicadae Periostracum] |
| 土鳖虫 | Eupolyphaga sinensis Walker | Eupolyphaga sinensis Walker [Corydiidae; Eupolyphaga] |
| 蜈蚣 | Scolopendra subspinipes mutilans L.Koch | Scolopendra subspinipes mutilans L.Koch [Scolopendridae; Scolopendra] |
| 檀香 | Santalum album L. | Santalum album L. [Santalaceae; Santali Albi Lignum] |
| 降香 | Dalbergia odorifera T.C.Chen | Dalbergia odorifera T.C.Chen [Fabaceae; Dalbergiae Odoriferae Lignum] |
| 乳香 | Boswellia carterii Birdw. | Boswellia carterii Birdw. [Burseraceae; Olibanum] |
| 酸枣仁 | Ziziphus jujuba Mill. var. spinosa (Bunge) Hu ex H.F.Chow | Ziziphus jujuba Mill. var. spinosa (Bunge) Hu ex H.F.Chow [Rhamnaceae; Ziziphi Spinosae Semen] |
| 冰片 | Dryobalanops aromatica C.F.Gaertn. | Dryobalanops aromatica C.F.Gaertn. [Dipterocarpaceae; Borneolum] |
| 桑白皮 | Morus alba L. | Morus alba L. [Moraceae; Mori Cortex] |
| 佛手 | Citrus medica L. var. sarcodactylis (Hoola van Nooten) Swingle | Citrus medica L. var. sarcodactylis (Hoola van Nooten) Swingle [Rutaceae; Citri Sarcodactylis Fructus] |
| 防风 | Saposhnikovia divaricata (Turcz.) Schischk. | Saposhnikovia divaricata (Turcz.) Schischk. [Apiaceae; Saposhnikoviae Radix] |
| 知母 | Anemarrhena asphodeloides Bunge | Anemarrhena asphodeloides Bunge [Asparagaceae; Anemarrhenae Rhizoma] |
| 浮小麦 | Triticum aestivum L. | Triticum aestivum L. [Poaceae; Tritici Levis Fructus] |
| 伊贝母 | Fritillaria pallidiflora Schrenk | Fritillaria pallidiflora Schrenk [Liliaceae; Fritillariae Pallidiflorae Bulbus] |
| 象贝母 | Fritillaria thunbergii Miq. | Fritillaria thunbergii Miq. [Liliaceae; Fritillariae Thunbergii Bulbus] |
| 沉香曲 | Aquilaria sinensis (Lour.) Spreng. | Aquilaria sinensis (Lour.) Spreng. [Thymelaeaceae; Aquilariae Lignum Resinatum] |
| 钟乳石 | Stalactitum | Stalactitum [Mineralia; Stalactitum] |
| 仙灵脾 | Epimedium brevicornu Maxim. | Epimedium brevicornu Maxim. [Berberidaceae; Epimedii Herba] |
| 枳壳 | Citrus × aurantium L. | Citrus × aurantium L. [Rutaceae; Aurantii Fructus] |
| 芝麻 | Sesamum indicum L. | Sesamum indicum L. [Pedaliaceae; Sesami Semen Nigrum] |
| 冰糖 | Saccharum | Saccharum [Poaceae; Saccharum] |
| 白芍 | Paeonia lactiflora Pall. | Paeonia lactiflora Pall. [Paeoniaceae; Paeoniae Radix Alba] |
| 白芥子 | Sinapis alba L. | Sinapis alba L. [Brassicaceae; Sinapis Albae Semen] |

Composition and Preparation Methods of Chinese Patent Medicines

| **Traditional Chinese Medicine drugs** | **Abbreviation** | **Composition** | **Chinese medicine formulations** | **Manufacturing operation** |
| --- | --- | --- | --- | --- |
| An-chuan-zhi-sheng ointment | ACZSO | Pseudostellariae radix  Rehmanniae radix  Ophiopogonis radix  Cornus officinalis  Achyranthis bidentatae radix  Lycii fructus  Schisandrae chinensis fructus  Juglans regia  Angelicae sinensis radix Magnetitum  Asteris radix et rhizoma  Farfarae flos  Pheretima  Aquilariae lignum resinatum  Asini corii colla  Cervus nippon Temminck | Ointment | After soaking the herbal ingredients according to the recipe, simmer them alternately with gentle and intense heat to concentrate and extract the medicinal properties. Then, add honey or syrup to thicken the mixture into a paste, continuously stirring until it reaches a consistency where a drop forms a pearl-like shape. Finally, while still warm, bottle and seal the product, which will solidify into a semi-fluid state upon cooling. |
| Fu-zheng-hua-zhuo ointment | FZHZO | Ficus hirta Vahl  Pseudostellariae radix  Atractylodis macrocephalae rhizoma Poria  Prunus armeniaca L. var. ansu Maxim.  Processed glue from turtle shell |  |  |
| Yi-fei ointment | YFO | Astragali radix  Codonopsis radix  Rehmanniae radix  Cornus officinalis  Schisandrae chinensis fructus  Polygonati rhizoma  Platycodi radix  Alismatis rhizoma  Mori cortex  Ophiopogonis radix  Citri sarcodactylis fructus  Prunus armeniaca L. var. ansu Maxim.  Salviae miltiorrhizae radix et rhizoma  Saposhnikoviae radix  Angelicae sinensis radix  Atractylodis macrocephalae rhizoma Poria  Dioscoreae rhizoma  Moutan Cortex  Citri reticulatae pericarpium  Glycyrrhizae radix et rhizoma  Asini corii colla  "Tortoise-shell glue  " |  |  |
| Zou-fei-ding-chuan ointment | ZFDCO | Astragali radix  Atractylodis macrocephalae rhizoma  Saposhnikoviae radix  Codonopsis radix  Poria  Glycyrrhizae radix et rhizoma  Rehmanniae radix  Corni fructus  Dioscoreae rhizoma  Moutan Cortex  Alismatis rhizoma  Salviae miltiorrhizae radix et rhizoma  Angelicae sinensis radix  Chuanxiong rhizoma  Paeoniae radix alba  Cinnamomi ramulus  Perilla frutescens* (L.) Britt. var. *frutescens  Semen brassicae  Ephedrae herba  Platycodi radix  Prunus armeniaca L. var. ansu Maxim.  Asteris radix et rhizoma  Farfarae flos  Pinelliae rhizoma  Citri reticulatae pericarpium  Mori cortex  Scutellariae radix  Fritillaria thunbergii  Aquilariae lignum resinatum  Schisandrae chinensis fructus  Stalactitum  "Herba epimedii  "  Psoraleae fructus  Polygonati rhizoma  Ophiopogonis radix  Aurantii fructus  Amomi fructus  Juglans regia L.  Sesami semen nigrum  Asini corii colla  Processed glue from turtle shell  Rock candy |  |  |
| Bai-ling capsule | BLC | Cordyceps | Capsule | First, the herbal materials are purified into powders or concentrated extracts. Automated filling equipment is then used to precisely fill the medicated powder or micro-granules into the two-piece gelatin capsule shells. Subsequently, the capsules are assembled and sealed to ensure accurate dosing. |
| Bu-fei-huo-xue capsule | BFHXC | Paeoniae radix rubra  Astragali radix  Psoraleae fructus |  |  |
| Shen-ge-yi-fei capsule | SGYFC | Panax ginseng  Gekko  Fritillaria cirrhosa  Glycyrrhizae radix et rhizoma  Notoginseng radix et rhizoma  Pheretima  Chuanxiong rhizoma |  |  |
| Gu-ben-ke-chuan capsule | GBKCC | Codonopsis radix  Atractylodis macrocephalae rhizoma  Poria  Ophiopogonis radix  Schisandrae chinensis fructus  Honey-fried Glycyrrhizae radix et rhizoma  Psoraleae fructus |  |  |
| Ge-jie-ding-chuan capsule | GJDCC | Trichosanthis semen  Gekko  Ephedrae herba  Asteris radix et rhizoma |  |  |
| Ke-chuan-ning capsule | KCNC | Rhododendron anthopogon D. Don  Scutellariae radix  Fritillariae thunbergii bulbus  Stemonae radix  Curcumae rhizoma  Epimedii herba |  |  |
| Jin-shui-bao capsule | JSBC | Cordyceps |  |  |
| Su-huang-zhi-ke capsule | SHZKC | Ephedrae herba  Perillae folium  Eriobotryae folium  Perilla frutescens* (L.) Britt. var. *frutescens  Pheretima |  |  |
| Tong-xin-luo capsule | TXLC | Ginseng radix Hirudo  Scorpio  Paeoniae radix rubra  Cicadae periostracum  Eupolyphaga, Steleophaga  Scolopendra  Santali albi lignum  Dalbergiae odoriferae lignum  Olibanum Ziziphi spinosae semen  Borneol |  |  |
| Yi-fei capsule | YFC | Prunus armeniaca L. var. ansu Maxim.  Mori cortex  Fritillaria cirrhosa  Panax ginseng  Gekko  Anemarrhenae rhizoma  Poria  Glycyrrhizae radix et rhizoma |  |  |
| Bu-fei-jian-pi granule | BFJPG | Astragali radix  Codonopsis radix  Atractylodis macrocephalae rhizoma  Poria  Fritillaria cirrhosa | Granule | Concentrate the decoction of medicinal materials into a thick paste, and mix it with excipients to form a soft material. Pass through mechanical extrusion and sieving to form wet granules, and then dry to remove moisture in a fluidized bed dryer. Finally, sieve out uniformly dried granules, and package them in bags. |
| Bu-fei-yi-shen granule | BFYSG | Ginseng radix  Astragali radix  Lycii fructus  Cornus officinalis  Epimedii herba |  |  |
| Yi-qi-zi-shen granule | YQZSG | Ginseng radix  Polygonati rhizoma  Rehmanniae radix  Ophiopogonis radix  Schisandrae chinensis fructus |  |  |
| Bu-fei-yi-yang-hua-tan granule | BFYYHTG | Pseudostellariae radix  Astragali radix  Eriobotryae folium  Asteris radix et rhizoma  Descurainiae semen, Lepidi semen  Schisandrae chinensis fructus  Fritillaria cirrhosa  Platycodi radix  Lycium chinense  Eucommiae cortex  Morindae officinalis radix |  |  |
| Bu-zhong-yi-qi granule | BZYQG | Astragali radix  Codonopsis radix  Glycyrrhizae radix et rhizoma  Atractylodis macrocephalae rhizoma  Angelicae sinensis radix  Jujubae fructus  Cimicifugae rhizoma  Bupleuri radix  Citri reticulatae pericarpium  Zingiberis rhizoma recens |  |  |
| Fei-kang granule | FKG | Astragali radix  Panacis quinquefolii radix  Poria  Cordyceps  Ganoderma  Salviae miltiorrhizae radix et rhizoma  Pinelliae rhizoma  Glycyrrhizae radix et rhizoma |  |  |
| Gu-ben-ke-chuan granule | GBKCG | Codonopsis radix  Atractylodis macrocephalae rhizoma  Poria  Ophiopogonis radix  Schisandrae chinensis fructus  Honey-fried Glycyrrhizae radix et rhizoma  Psoraleae fructus |  |  |
| Ping-chuan-yi-qi granule | PCYQG | Ephedrae herba  Panax ginseng  Dioscoreae nipponicae rhizoma  Prunus armeniaca L. var. ansu Maxim.  Magnoliae officinalis cortex  Citri reticulatae pericarpium  Glycyrrhizae radix et rhizoma  Bupleuri radix  Perillae folium |  |  |
| Yi-fei-huo-xue granule | YFHXG | Astragali radix  Codonopsis radix  Paeoniae radix rubra  Chuanxiong rhizoma |  |  |
| Yi-qi-jian-pi granule | YQJPG | Codonopsis radix  Atractylodis macrocephalae rhizoma  Poria  Dioscoreae rhizoma  Citri reticulatae pericarpium  Perillae caulis  Honey-fried Glycyrrhizae radix et rhizoma |  |  |
| Yu-ping-feng granule | YPFG | Atractylodis macrocephalae rhizoma  Astragali radix  Saposhnikoviae radix |  |  |
| Shen-ling-bai-zhu powder | SLBZP | Lablab semen album  Atractylodis macrocephalae rhizoma  Poria  Glycyrrhizae radix et rhizoma  Platycodi radix  Nelumbinis semen  Ginseng radix  Amomi fructus  Dioscoreae rhizoma  Coicis semen | Powder | The dry medicinal materials are repeatedly ground using a pulverizer, passed through a fine sieve to achieve extremely fine powder. They are strictly mixed according to the formula and sterilized if necessary. |
| Jia-wei-shen-ge powder | JWSGP | Ginseng radix  Gekko  Astragali radix  Honey-fried Glycyrrhizae radix et rhizoma  Poria  Schisandrae chinensis fructus  Reineckia carnea (Andr.) Kunth  Cordyceps  Hominis placenta |  |  |
| Gu-shen-ding-chuan pill | GSDCP | Rehmanniae radix  Processed product from Aconitum carmichaelii Debx  Moutan Cortex  Achyranthis bidentatae radix  Psoraleae fructus  Amomi fructus  Plantaginis semen  Poria  Alpinia oxyphylla Miq. Cinnamomi cortex  Dioscoreae rhizoma  Alismatis rhizoma  Rosae laevigatae fructus | Pill | Honey pills are made using refined honey as a binder, mixed with medicinal powder, formed into strips, and then handcrafted or mechanically divided and rolled into pills. Water pills are made by utilizing the inherent adhesiveness of the medicinal powder to form pills. |
| Jin-kui-shen-qi pill | JKSQP | Aconiti radix lateralis praeparata  Cinnamomi ramulus  Rehmanniae radix  Corni fructus  Dioscoreae rhizoma  Alismatis rhizoma  Poria  Root bark of the peony tree |  |  |
| Yi-qi-gu-biao pill | YQGBP | Codonopsis radix  Atractylodis macrocephalae rhizoma  Poria  Citri reticulatae pericarpium  Pinelliae rhizoma  Coicis semen  "Fructus Tritici Levis  "  Perilla frutescens* (L.) Britt. var. *frutescens  Farfarae flos  Scutellariae radixFritillaria pallidiflora Schrek.  Eriobotryae folium  Saposhnikoviae radix |  |  |
| San-ao tablet | SAT | Ephedrae herba  Prunus armeniaca L. var. ansu Maxim.  Glycyrrhizae radix et rhizoma | Tablet | The powder is granulated with disintegrants, binders and other excipients, and then compressed into tablets at high speed using a tablet press. Some require coating to improve taste or control release. |
