# Supplementary material for: Efficacy and safety of oral proprietary Chinese medicines in the treatment of stable chronic obstructive pulmonary disease: a network meta-analysis
Source: Front Pharmacol. 2026 Jan 21;16:1690739. doi: 10.3389/fphar.2025.1690739 (PMC12868190; doi:10.3389/fphar.2025.1690739)
Supplement: Supplementary file 4 [file Table5.docx]

**Supplementary Document 5** Publication bias


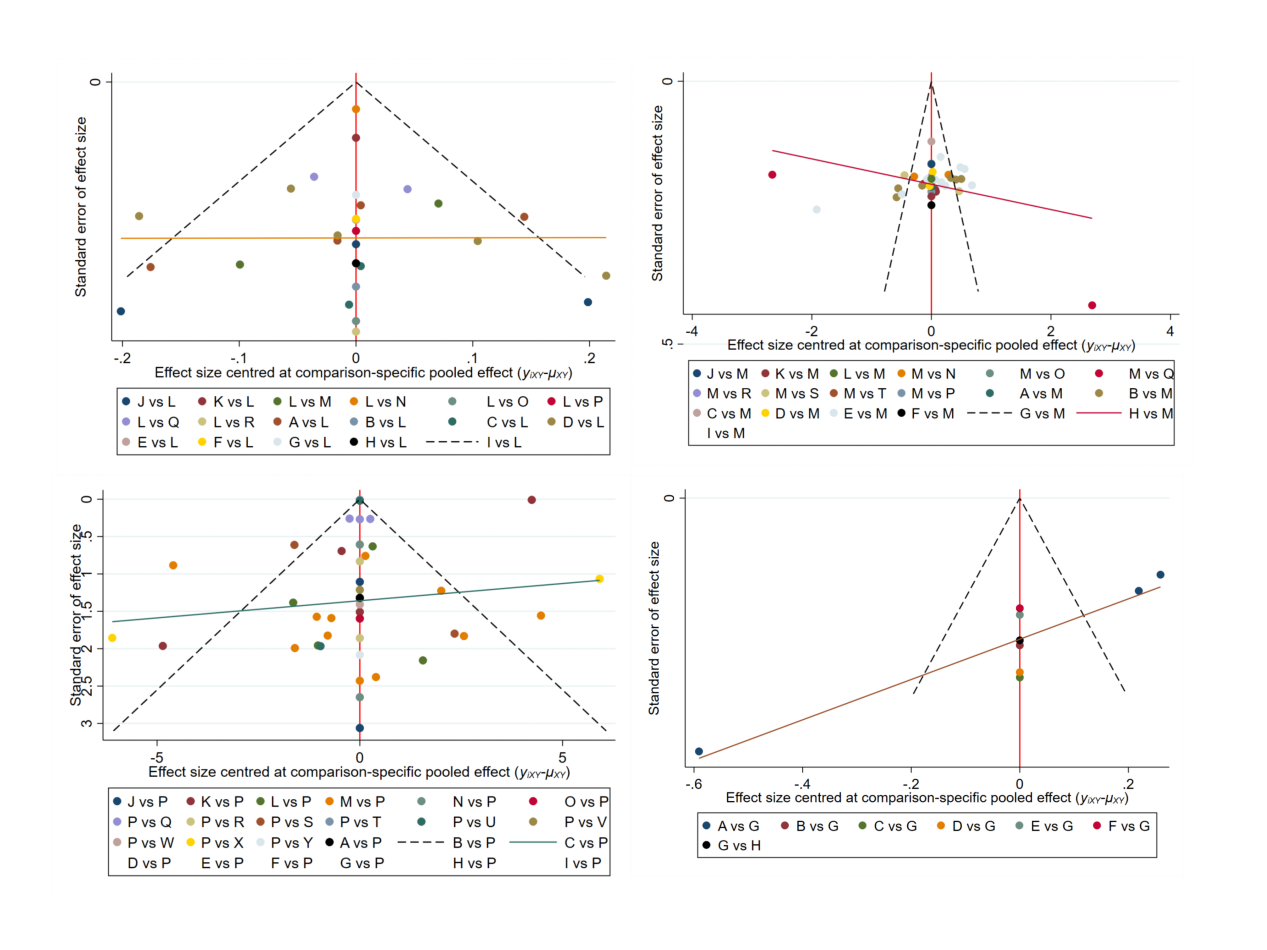


Figure S7 Comparison-adjusted funnel plots. A: FVC; B: FEV_1_; C: FEV_1_/FVC ratio; D: PEF.


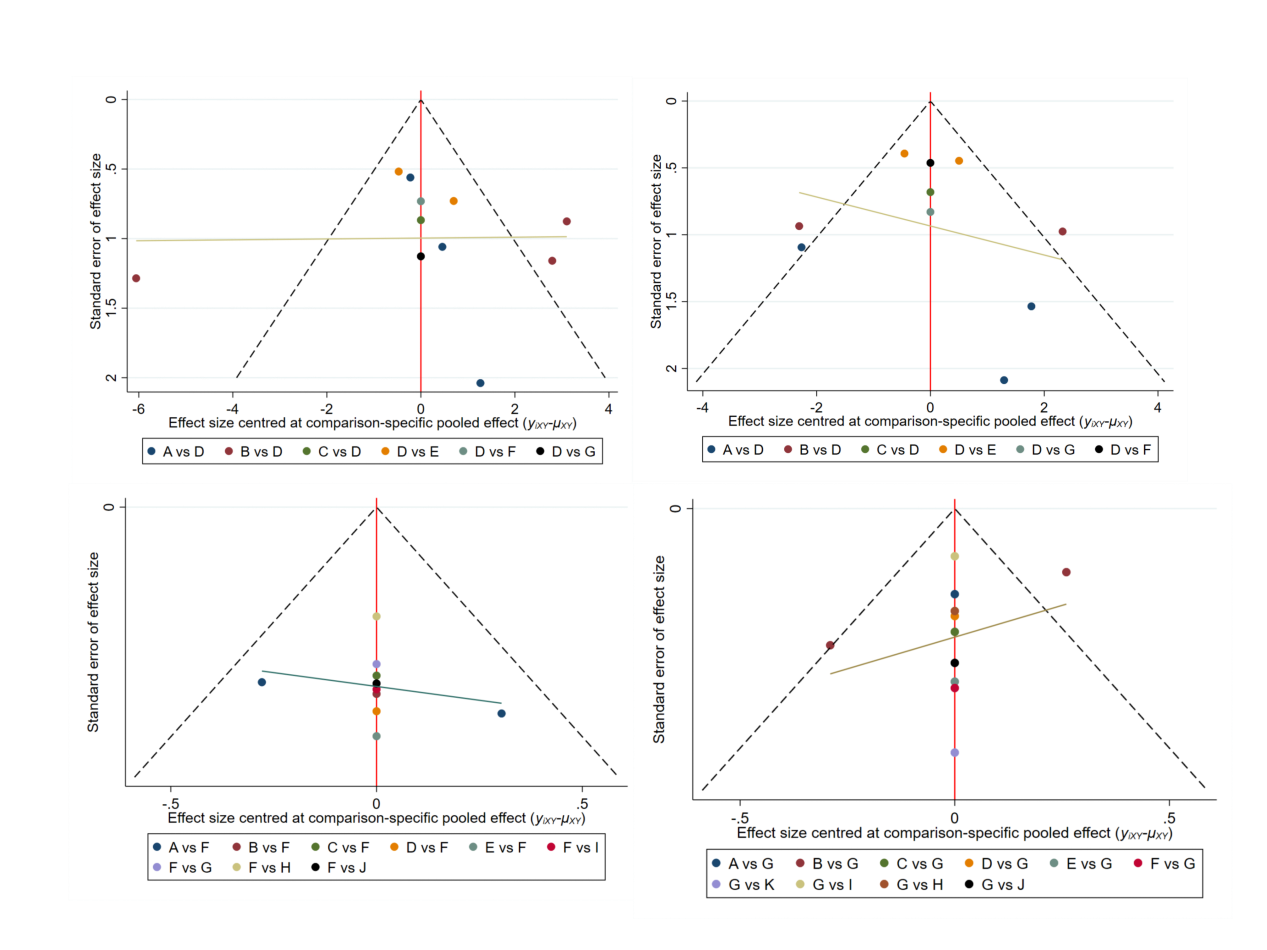


Figure S8 Comparison-adjusted funnel plots. A: PaO_2_; B: PaCO_2_; C: TNF-α; D: mMRC.


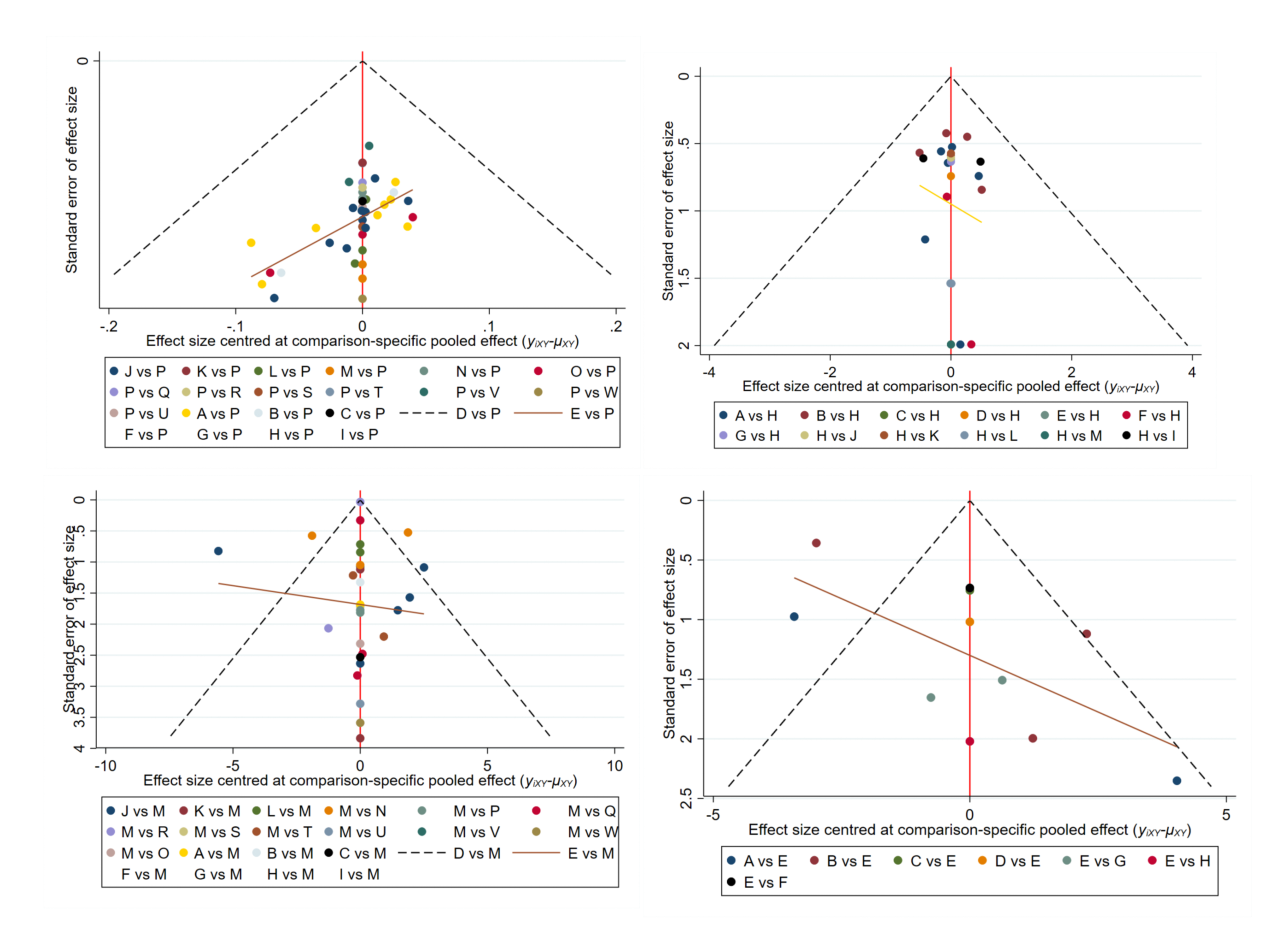


Figure S9 Comparison-adjusted funnel plots. A: total effective rate; B: adverse reactions; C: FEV_1_%; D: SGRQ.


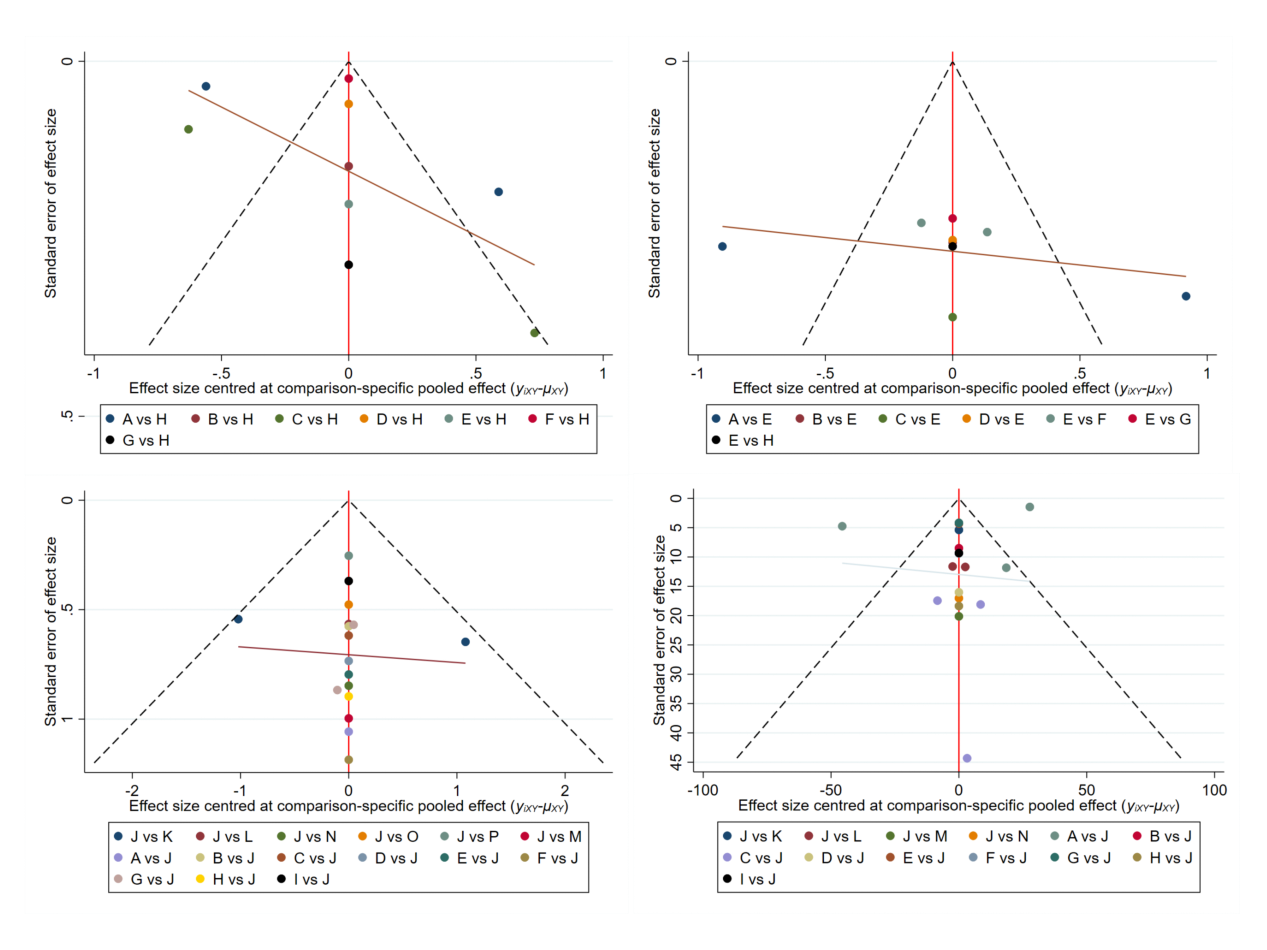


Figure S10 Comparison-adjusted funnel plots. A: number of acute exacerbations; B: IL-8; C: CAT; D: 6MWD.
