# Supplementary material for: Efficacy and safety of oral proprietary Chinese medicines in the treatment of stable chronic obstructive pulmonary disease: a network meta-analysis
Source: Front Pharmacol. 2026 Jan 21;16:1690739. doi: 10.3389/fphar.2025.1690739 (PMC12868190; doi:10.3389/fphar.2025.1690739)
Supplement: Supplementary file 5 [file Table3.docx]

**Supplementary Document 3** Detailed Methodological Appendix

Network Meta-Analysis for "Efficacy and safety of oral proprietary Chinese medicines in the treatment of stable chronic obstructive pulmonary disease: a network meta-analysis"

Date of Analysis:May 30, 2024.

Software Used: R software (version 4.1.3)

Packages Used: gemtc package (version 1.0-1), JAGS software

**1. Data Structure and Preparation**

The analysis dataset was structured in a one-row-per-arm format, containing the following key variables for each outcome:

For the data reported by WMD:

study: Unique identifier for each study.

treatment: Numeric identifier for each intervention.

sampleSize: Number of participants in the arm.

mean: Mean outcome score.

std.dev: Standard deviation of the outcome score.

For the data reported by SMD:

study: Unique identifier for each study.

treatment: Numeric identifier for each intervention.

diff: standardized mean difference.

std.err: The corresponding standard error.

For the data reported by RR:

study: Unique identifier for each study.

treatment: Numeric identifier for each intervention.

sampleSize: Number of participants in the arm.

responders: The number of people whose outcome occurred.

**2. R Scripts for Network Meta-Analysis (FVC)**

The following provides a representative script used to conduct the Bayesian network meta-analysis for the primary outcome of FVC (MD). Similar scripts were used for the other outcomes (SMD).

code

R

// *****************************************************************************

//

setwd('C:/Users/Administrator/Desktop/FVC ')

install.packages('gemtc')

library(gemtc)

FVC <- read.csv('FVC.csv',head = T)

network <- mtc.network(FVC) #MD ;( network <- mtc.network(data.re = (FVC) #SMD)

plot(network)

model <-mtc.model(network, type="consistency", n.chain=4,likelihood="normal",link="identity",linearModel="random")

results <- mtc.run(model, n.adapt = 20000, n.iter = 50000, thin = 1)

summary(results)

modelume <-mtc.model(network, type="ume", n.chain=4,likelihood="normal",link="identity",linearModel="random")

resultsume <- mtc.run(modelume, n.adapt = 20000, n.iter = 50000, thin = 1)

summary(resultsume)

forest(relative.effect(results, "RT"),digits=3)

ranks <- rank.probability(results)

sucra(ranks)

a <- relative.effect.table(results)

write.csv(a1, "FVC_table.csv")

==============================================================================

**3. R Scripts for Network Meta-Analysis (Total effective rate)**

The following provides a representative script used to conduct the Bayesian network meta-analysis for the primary outcome of Total effective rate (RR).

code

R

// *****************************************************************************

//

setwd('C:/Users/Administrator/Desktop/TER')

install.packages('gemtc')

library(gemtc)

TER <- read.csv('TER.csv',head = T)

network <- mtc.network(TER)

plot(network)

model <- mtc.model(network, type="consistency", n.chain=4, likelihood = "binom", link="log", linearModel = "random")

results <- mtc.run(model, n.adapt = 20000, n.iter = 50000, thin = 1)

summary(results)

modelume <-mtc.model(network, type="ume", n.chain=4,likelihood="binom",link="log",linearModel="random")

resultsume <- mtc.run(modelume, n.adapt = 20000, n.iter = 50000, thin = 1)

summary(resultsume)

forest(relative.effect(results, "RT"),digits=3)

ranks <- rank.probability(results)

sucra(ranks)

a <- relative.effect.table(results)

write.csv(a1, "TER _table.csv")

==============================================================================

SUMMARY OF INCONSISTENCY TESTS

==============================================================================

The DIC difference for the FVC outcome was 0.18018, while for the Total effective rate outcome, the DIC difference was 0.35883; the differences for all other outcomes were less than 5.This indicates that there is no statistical evidence of inconsistency between the direct and indirect evidence for any comparison within the network. Therefore, the consistency assumption holds, and the network meta-analysis results are considered reliable.

**4. Conclusion of Methodological Appendix**

The analyses were conducted following the pre-specified protocol. Both the transitivity assumption (assessed qualitatively) and the consistency assumption (assessed statistically) were judged to be valid for all outcome networks. The provided scripts and log outputs confirm the methodological transparency and reproducibility of the findings presented in the main manuscript.
