# Supplementary material for: Efficacy and safety of oral proprietary Chinese medicines in the treatment of stable chronic obstructive pulmonary disease: a network meta-analysis
Source: Front Pharmacol. 2026 Jan 21;16:1690739. doi: 10.3389/fphar.2025.1690739 (PMC12868190; doi:10.3389/fphar.2025.1690739)
Supplement: Supplementary file 6 [file Table4.docx]

**Supplementary Document 4** Other outcome indicators

**3.4.11 Secondary outcome indicators**

**3.4.11.1 FEV_1_%**

29 studies involving 4,049 participants investigated FEV_1_% [1-29]. In addition to RT, 22 OPCMs were included: BLC, BLC_BFHXC, BFHXC, BFJPG_BFYSG_YQZSG, BZYQG, SGYFC, SLBZP, GBKCG, GSDCP, KCNC, JWSGP, JKSQP, JSBC, JSBC_BFHXC, PCYQG, SAT, TXLC, YFP, YFHXG, YQJPG, YPFG, and ZFDCP. The network plot illustrating the various interventions is presented in Figure S1A. The findings indicated that compared to PCYQG (MD=13.62, 95% CrI: 4.91, 22.16) and RT (MD=8.47, 95% CrI: 4.44, 12.35), BLC performed better in improving FEV_1_% (Figure S1B). According to SUCRA, BLC (SUCRA: 80%), JKSQP (SUCRA: 72.3%), and JWSGP (SUCRA: 70.2%) may be the best three measures to improve FEV_1_% (Figure S1C).

**3.4.11.2 SGRQ**

11 studies involving 1,649 participants examined SGRQ [1, 4, 9, 10, 12, 16, 20, 30-33]. In addition to RT, seven OPCMs were included: BLC_BFHXC, SHZKC, BLC, BFHXC, GSDCP, YFHXG, and YFP. The network plot illustrating the various interventions is presented in Figure S2A. The results revealed that BFHXC (MD=-9.54, 95% CrI: -17.78, -2.2) and BLC (MD=-7.22, 95% CrI: -13.62, -1.15) may be superior to RT in improving SGRQ scores, with statistically significant differences. Other pairwise interventions implied no statistically significant differences (Figure S2B). According to SUCRA, BFHXC (SUCRA: 81.6%), YFP (SUCRA: 74.6%), and GSDCP (SUCRA: 63.5%) may be the three most effective interventions in improving SGRQ scores (Figure S2C).

**3.4.11.3 Number of acute exacerbations**

Nine studies involving 1,327 participants reported this outcome indicator [1, 6, 21, 30, 34-38]. In addition to RT, seven OPCMs were included: BFHXC, BLC, BFJPG_BFYSG_YQZSG, BLC_BFHXC, FZHZP, JKSQP, and JSBC_BFHXC. The network plot illustrating the various interventions is presented in Figure S3A. The results demonstrated that all pairwise interventions implied no statistically significant differences (Figure S3B). According to SUCRA, JSBC_BFHXC (SUCRA: 77.6%), BLC (SUCRA: 72.5%), and BFHXC (SUCRA: 62.3%) may represent the three most effective interventions for reducing the number of acute exacerbations (Figure S3C).

**3.4.11.4 IL-8**

Nine studies involving 1,126 participants reported IL-8 [9, 11, 12, 25, 30, 39-42]. In addition to RT, seven OPCMs were included: BLC, GBKCG, GSDCP, JSBC, SHZKC, YQGBP, and ZFDCP. The network plot illustrating the various interventions is presented in Figure S4A. The results indicated that compared to RT (MD=2.59, 95% CrI: 0.77, 4.41), GSDCP demonstrated superior efficacy in improving IL-8 levels (Figure S4B). According to SUCRA, GSDCP (SUCRA: 91.5%), BLC (SUCRA: 73.4%), and YQGBP (SUCRA: 57.9%) may represent the three most effective interventions for reducing IL-8 levels (Figure S4C).

**3.4.11.5 CAT**

17 studies involving 2,460 participants investigated CAT [2, 10, 13, 14, 17, 18, 20, 22, 25, 35, 40, 43-48]. In addition to RT, 15 OPCMs were included: ACZSO, BLC, FKG, BZYQG, GBKCC, KCNC, JKSQP, JSBC, PCYQG, SAT, SHZKC, YFP, YFC, YQGBP, and YPFG. The network plot illustrating the various interventions is presented in Figure S5A. The results implied that ACZSO (MD=-7.21, 95% CrI: -13.62, -0.74) and PCYQG (MD=-8.01, 95% CrI: -14.25, -1.75) may be superior to RT in improving CAT, with statistically significant differences (Figure S5B). According to SUCRA, PCYQG (SUCRA: 95.7%), ACZSO (SUCRA: 89.9%), and YFC (SUCRA: 88.3%) be the three most effective interventions in improving CAT (Figure S5C).

**3.4.11.6 6MWD**

18 studies involving 2,334 participants explored this outcome indicator [11, 13-16, 18, 20, 26, 33, 36-38, 43, 45, 48-51]. In addition to RT, 13 OPCMs were included: BLC, BFHXC, BFJPG_BFYSG_YQZSG, BZYQG, GBKCC, GJDCC, KCNC, JSBC, PCYQG, YFHXG, YFC, YQJPG, and ZFDCP. The network plot illustrating the various interventions is presented in Figure S6A. The results demonstrated that all pairwise interventions implied no statistically significant differences (Figure S6B). According to SUCRA, PCYQG (SUCRA: 88.2%), BLC (SUCRA: 70.7%), and BFHXC (SUCRA: 67.1%) may represent the three most effective interventions for improving 6MWD (Figure S6C).


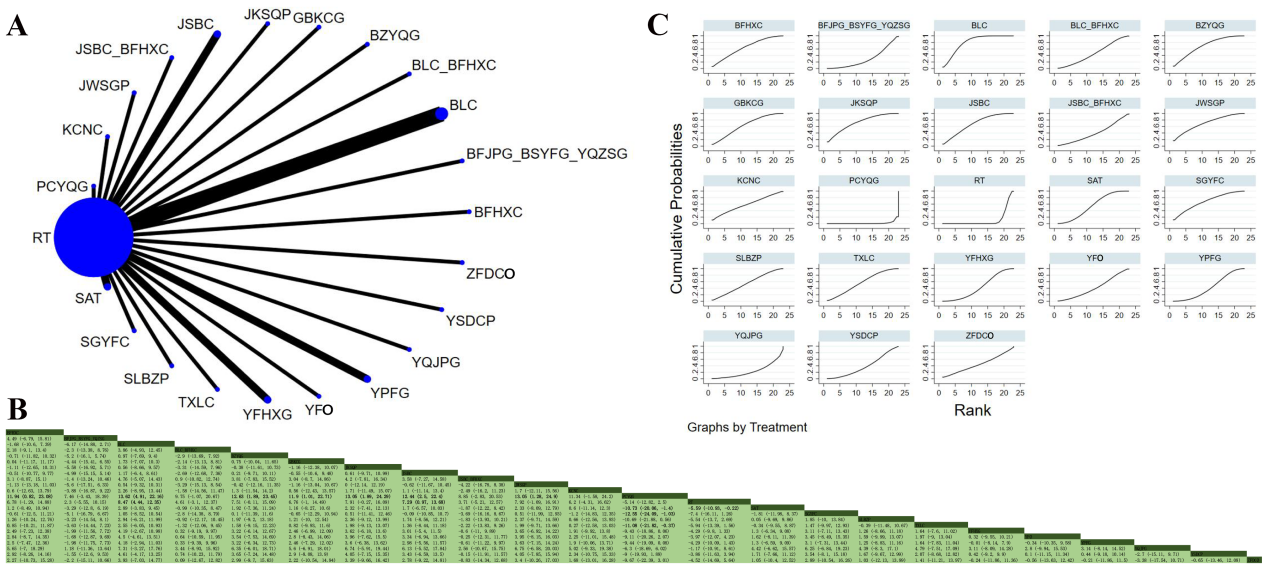


Figure S1 Network plot and network meta-analysis results. (A) Network plot for FEV_1_%; (B) Relative effect of different OPCMs on FEV_1_%; (C) Cumulative probability line graph.


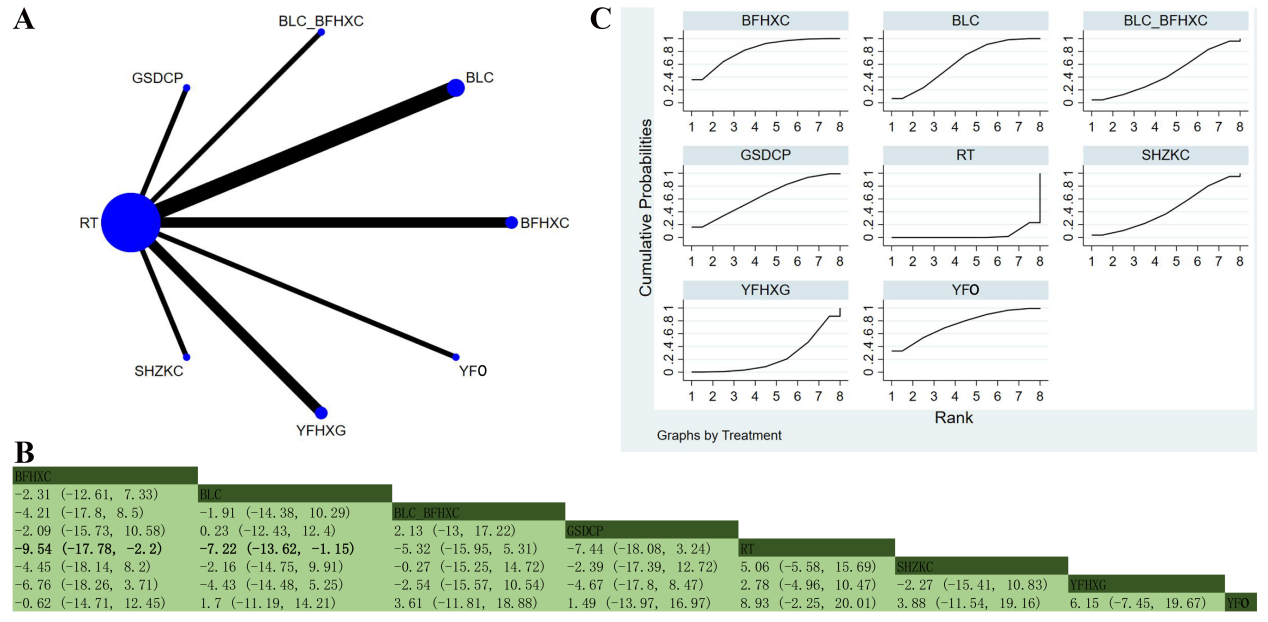


Figure S2 Network plot and network meta-analysis results. (A) Network plot for SGRQ; (B) Relative effect of different OPCMs on SGRQ; (C) Cumulative probability line graph.


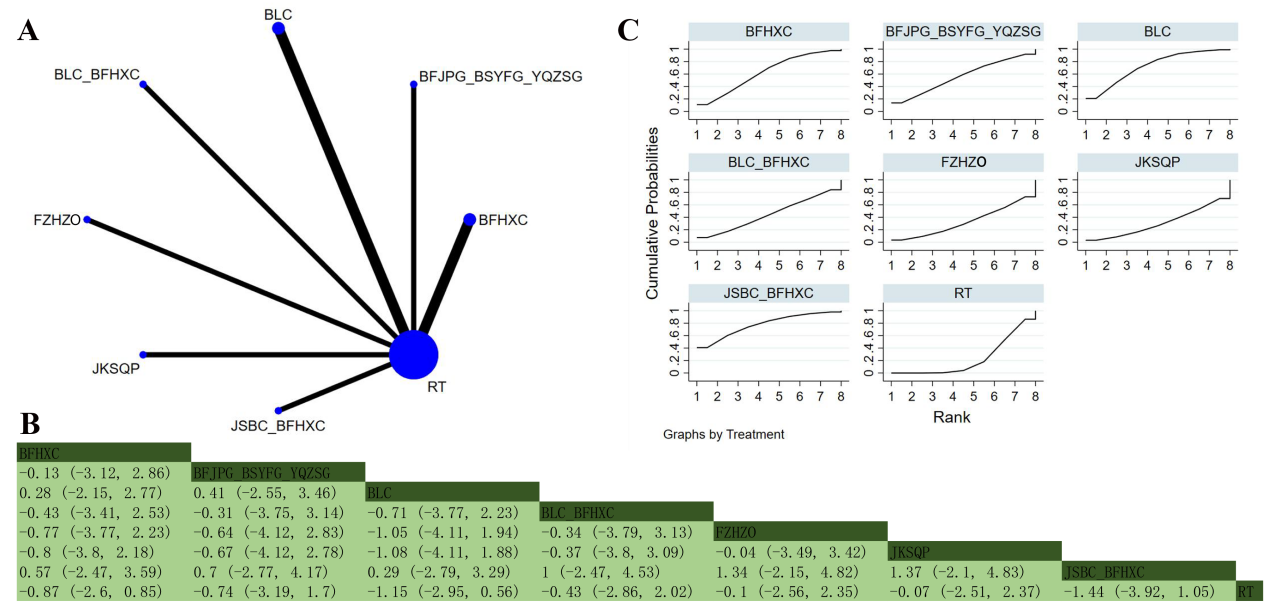


Figure S3 Network plot and network meta-analysis results. (A) Network plot for number of acute exacerbations; (B) Relative effect of different OPCMs on the number of acute exacerbations; (C) Cumulative probability line graph.


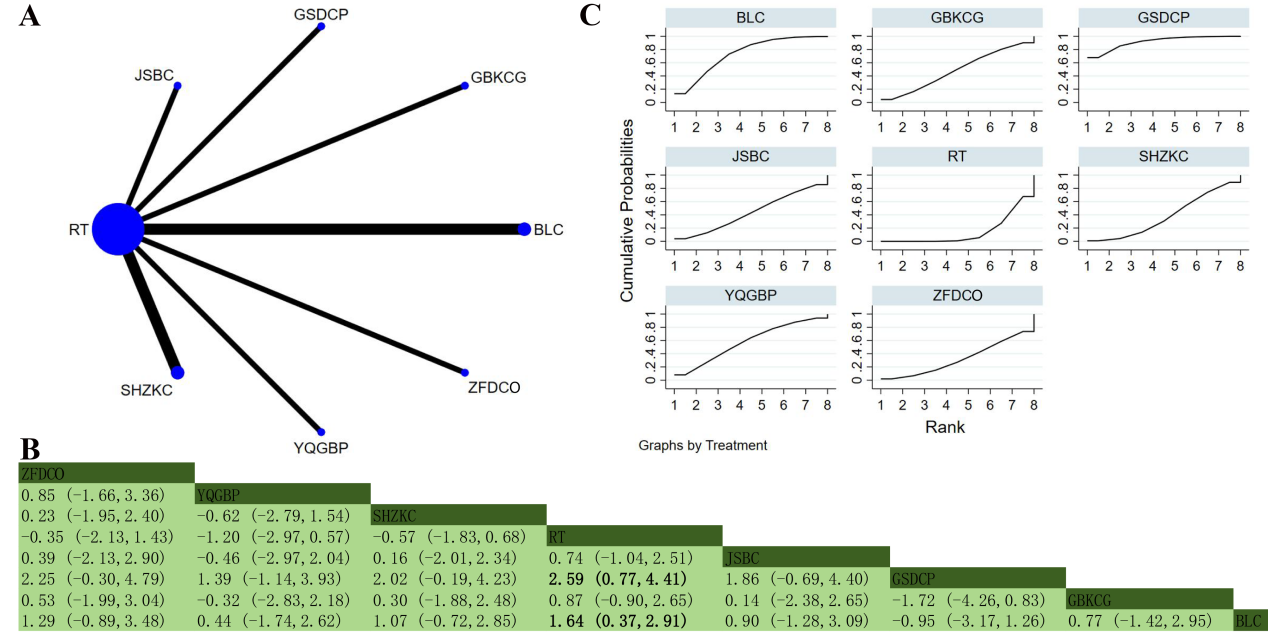


Figure S4 Network plot and network meta-analysis results. (A) Network plot for IL-8; (B) Relative effect of different OPCMs on IL-8; (C) Cumulative probability line graph.


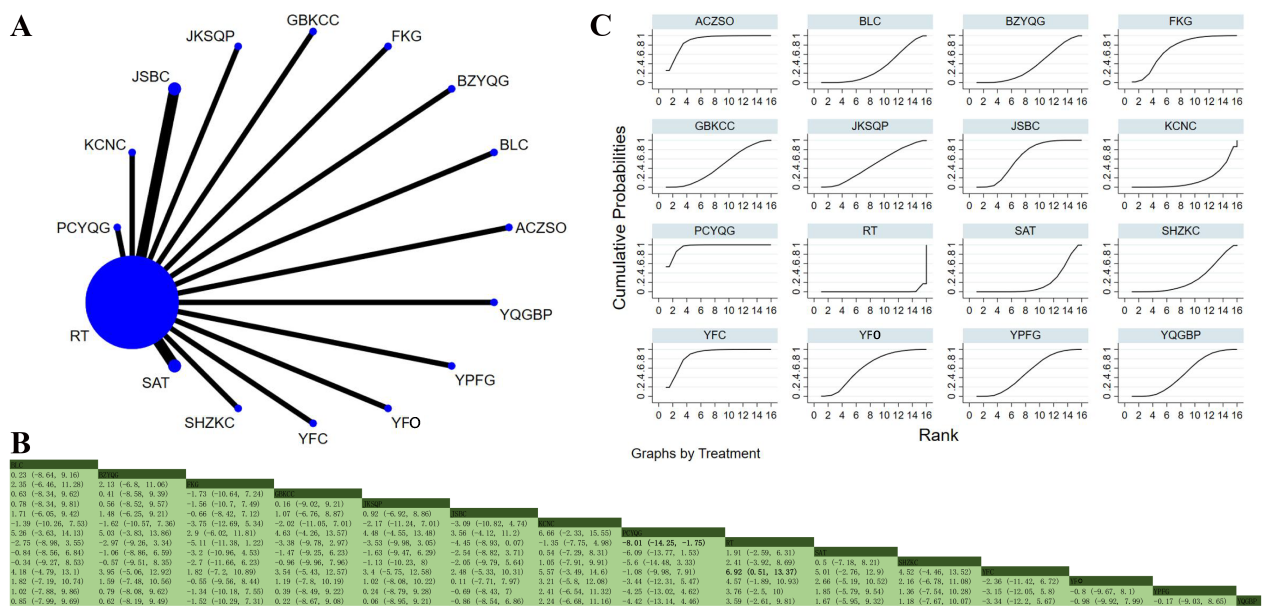


Figure S5 Network plot and network meta-analysis results. (A) Network plot for CAT; (B) Relative effect of different OPCMs on CAT; (C) Cumulative probability line graph.


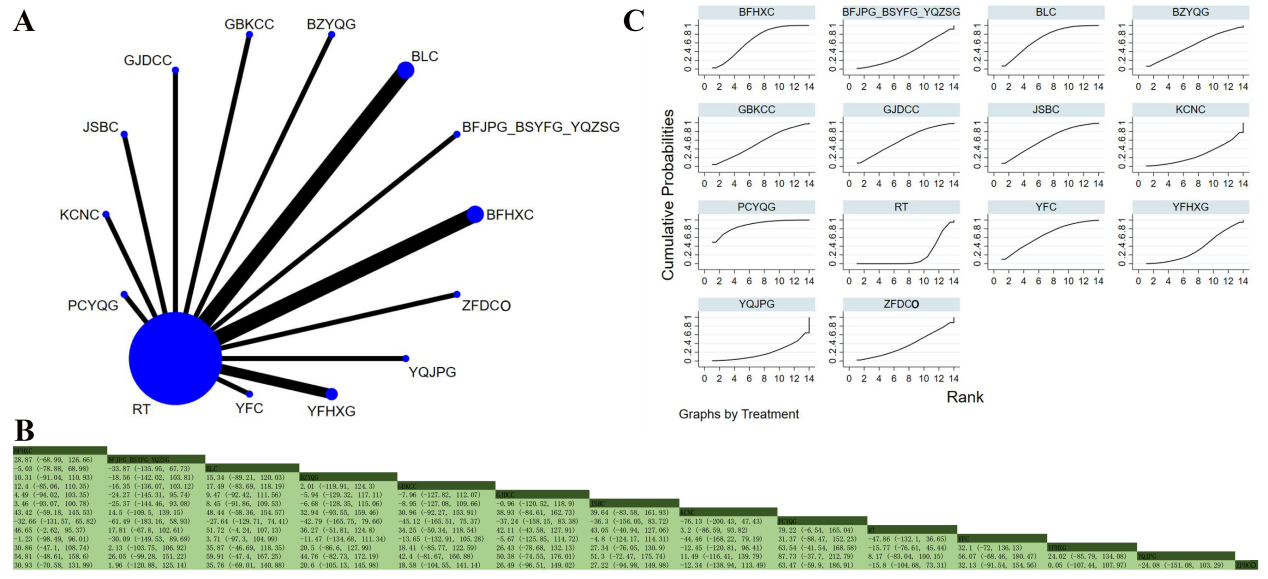


Figure S6 Network plot and network meta-analysis results. (A) Network plot for 6MWD; (B) Relative effect of different OPCMs on 6MWD; (C) Cumulative probability line graph.

**References**

1. Bai SR, Wu Y, Wamg Y, Liu J, Li P, Shen L*, et al.* Effect of Bailing Capsules Combined with Bufei Huoxue Capsules on Pulmonary Rehabilitation in Patients with Chronic Obstructive Pulmonary Disease at Stable Stage of Lung-kidney-Qi Deficiency Syndrome. Chinese Journal of Experimental Traditional Medical Formulae. 2016; 22(24):182-6. doi:10.13422/j.cnki.syfjx.2016240182.

2. Chen J, Zhu D, Chen H, Yuan F, Wamg LY, Xu JL. Clinical Study on Yupingfeng Granules Combined with Acetylcysteine for Chronic Obstructive Pulmonary Disease with Lung Qi Deficiency Syndrome at Stable Stage. Journal of New Chinese Medicine. 2021; 53(15):20-4. doi:10.13457/j.cnki.jncm.2021.15.005.

3. Yi X, Wang JJ, Liu W, Ge ZX. Effects of "JiaweiShenge Powder" for Treating Patients with Early Renal Damage and Chronic Obstructive Pulmonary Disease at Stable Phrase (Lung and Kidney Qi Dificiency). Asia-Pacific Traditional Medicine. 2015; 11(5):112-4. doi:10.11954/ytctyy.201505059.

4. Hao WD, Wang GF, Zhang CL. Efficacy and mechanism of Bailing capsule combined with budesonide and formoterol fumarate powder in the treatment of stable COPD patients. Journal of Clinical Pulmonary Medicine. 2016; 21(09):1603-6. doi:10.3969/j.issn.1009-6663.2016.09.015.

5. Peng D, Zhang YX, Gao JQ, Hao WD. Effect of Jin Shui Bao capsule combined with AVAPS ventilation in the treatment of stable chronic obstructive pulmonary disease. Modern Journal of Integrated Traditional Chinese and Western Medicine. 2018; 27(20). doi:10.3969/j.issn.1008-8849.2018.20.004.

6. Q.G. H, Liu LL. Clinical observation of Jinshuibao capsule combined with tonifying lung and activating blood capsule in the treatment of chronic obstructive pulmonary disease in stable stage Journal of Guiyang College of Traditional Chinese Medicine. 2012; 34(1):135-7. doi:10.3969/j.issn.1002-1108.2012.01.72.

7. Jiang MZ, Fei HY, Wang QB, Peng JW, Jiang J, Yang Q*, et al.* Curative effect and prognosis analysis of Tongxinluo capsule on patients with chronic obstructive pulmonary disease at remission. Journal of Clinical and Experimental Medicine. 2017; 16(15):1476-80. doi:10.3969/j.issn.1671-4695.2017.15.007.

8. Huang XQ, Guo B, Zhong HW, Gu YY, Chen H. Clinical Study of Shenge Yifei Capsule on Chronic Obstructive Pulmonary Disease. Research of Integrated Traditional Chinese and Western Medicine. 2021; 13(6):361-4,8. doi:10.3969/j.issn.1674-4616.2021.06.001.

9. Gui K, Yang J, Long QZ, Huang Y. Efficacy of Gushen Dingchuan Pills and Western Medicine in Treating Stable Chronic Obstructive Pulmonary Disease Patients with Syndrome of Lung and Kidney Qi Deficiency. Chinese Journal of Experimental Traditional Medical Formulae. 2019; 25(8):89-94. doi:10.13422/j.cnki.syfjx.20190731.

10. Jia JH, Zhou Q. Effects of the Bailing capsules plus umeclidinium bromide and vilanterol powder inhalation on stable chronic obstructive pulmonary disease (the lung and kidney both deficiency syndrome) and its influence on quality of life. Clinical Journal of Chinese Medicine. 2022; 14(25):105-9. doi:10.3969/j.issn.1674-7860.2022.25.030.

11. Ju Y, Xu JH, J.W. C, Ouyang BS, Wang DS, Tang L*, et al.* Effect of Zhoufei Dingchuan Ointment on BODE Index, IL-8 and TNF-α in Patients with Chronic Obstructive Pulmonary Disease. Chinese Journal of Hemorheology. 2021; 31(4):506-9,36. doi:10.3969/j.issn.1009-881X.2021.04.015.

12. Li L, Xie Z, Liao M. Clinical study on Guben Kechuan Granules combined with salbutamol in treatment of stable phase of chronic obstructive pulmonary disease. Drugs & Clinic. 2019; 34(06):1731-4. doi:10.7501/j.issn.1674-5515.2019.06.027.

13. Liu XQ, Xie WY. Fifty-Eight Cases of Senile Stable Chronic Obstructive Pulmonary Disease Treated with Pingchuan Yiqi Keli. Henan Traditional Chinese Medicine. 2015; 35(8):1997-8. doi:10.16367/j.issn.1003-5028.2015.08.0839.

14. Ma YF, Fan BX, Xu JE, Li C, Zhao WJ, Li GL*, et al.* Therapeutic efficacy of tonifying medium and benefiting qi granules in the treatment of moderate-to-severe chronic obstructive pulmonary disease in the stable stage of chronic obstructive pulmonary disease. Guiding Journal of Traditional Chinese Medicine and Pharmacology. 2015; 21(8):67-9.

15. Ou M, Zhang C, Song XJ, Wu N. Study of Yifei Huoxue granules on patients with moderately severe chronic obstructive pulmonary disease (COPD). Chinese Journal of Health Care and Medicine. 2014; 16(4):280-2. doi:10.3969/.issn.1674-3245.2014.04.009.

16. Ou M, Zhang C, Song XJ, Wu N. Effects of Yifei Huoxue granule on quality of life and pulmonary function in patients with stable chronic obstructive pulmonary disease. Journal of Beijing University of Traditional Chinese Medicine. 2015; 38(3):206-8,11. doi:10.3969/j.issn.1006-2157.2015.03.013.

17. Shangguan H, Dong L. Clinical effects of sanao tablets combined with western medicine to treat the stable chronic obstructive pulmonary disease. Journal of Clinical Internal Medicine. 2015; 32(2):100-2. doi:10.3969/j.issn.1001-9057.2015.02.009.

18. Sun J, Han MJ, Zhang XH, Cao PP. Random-controlled Trial of Kechuanning Capsules on Chronic Obstructive Pulmonary Disease Patients. Chinese Journal of Information on Traditional Chinese Medicine. 2014; (7):22-4. doi:10.3969/j.issn.1005-5304.2014.07.007.

19. Wang HT. The efficacy of Symbicort combined with Bailing Capsules in the treatment of chronic obstructive pulmonary disease in the stable stage of chronic obstructive pulmonary disease Guide of China Medicine. 2018; 16(19):120-1.

20. Wang MJ, Du T, Song H, Zhao LM. Comprehensive evaluation of Yifei plaster combined with tiotropium bromide in the treatment of patients with stable chronic obstructive pulmonary disease. Clinical Journal of Chinese Medicine. 2022; 14(3):48-51. doi:10.3969/j.issn.1674-7860.2022.03.016.

21. Wang MH, Li SY, Li JS, Yu XQ. A Validation Study on Clinical Evaluation Index System for Chronic Obstructive Pulmonary Disease Stabilization Period. Journal of Traditional Chinese Medicine. 2013; 54(20).

22. Wang YP, Yan W, Sun H. Observation of clinical efficacy on stable moderate and severe COPD patients treated with Sanaopian Combined with Seretide. Journal of Traditional Chinese Medicine University of Hunan. 2013; 33(10):27,59. doi:10.3969/j.issn.1674-070X.2013.10.018.027.02.

23. Wang YR, Bai GH. Observations on the efficacy of lung tonic and blood circulation capsule in the treatment of patients with chronic obstructive pulmonary disease in the stabilized stage. Healthmust-Readmagazine. 2019; (35):54.

24. Zhang J, Ma YT, Wang J, Wei WG. Clinical study on Jinkui Shenqi Pills combined with Salmeterol Xinafoate and Fluticasone Propionate Powder for inhalation in treatment of stable stage of chronic obstructive pulmonary disease. Drugs & Clinic. 2020; 35(3):455-9. doi:10.7501/j.issn.1674-5515.2020.03.013.

25. Zhuang L, Liang J, Zhang H, Wu JH. Clinical study on Jinshuibao Capsules combined with carboxymethylsteine in treatment of stable phase of COPD. Drugs & Clinic. 2019; 34(10):2975-9. doi:10.7501/j.issn.1674-5515.2019.10.019.

26. Yang JC, Wang Z, Lou YF, Xu JP, Hong HH, R.L. C*, et al.* Clinical Observation on Qi-Boosting Spleen-Fortifying Granules Combined with Western Medicine for 60 Cases of Stable Chronic Obstructive Pulmonary Disease with Lung-Spleen Qi Deficiency Pattern. Journal of Traditional Chinese Medicine. 2013; 54(22).

27. Zhai XM, Yuan XM. Therapeutic effect of budesonide formoterol combined with Bailing capsule in the treatment of elderly patients with stable COPD. Journal of Clinical Psychosomatic Diseases. 2019; 25(5):144-6. doi:10.3969/j.issn.1672-187X.2019.05.038.

28. Zhang LF, Duan B, Liu F, Tian XZ, Zhang YF. Effect and Mechanism of Shenling Baizhu Powder in Treatment of Stable Phase of Chronic Obstructive Pulmonary Disease. Liaoning Journal of Traditional Chinese Medicine. 2023; 50(12):109-12. doi:10.13192/j.issn.1000-1719.2023.12.031.

29. Wu SB, Li H. Clinical effect of Yupingfeng granules in the treatment of stable chronic obstructive pulmonary disease. Chinese and Foreign Medical Research. 2018; 16(36):126-7. doi:10.14033/j.cnki.cfmr.2018.36.059.

30. Cheng DZ, Du WF, Wu YM. Effect of Suhuang Zhike Capsules combined with Montelukast in the treatment of patients with stable chronic obstructive pulmonary disease. China Medical Herald. 2020; 17(4):163-6,70.

31. Guo J, Wu L, Tian ZF, Dong X, Jia L, Liu M*, et al.* Therapeutic efficacy of lung tonic and blood circulation capsule in treating stabilized COPD patients Modern Journal of Integrated Traditional Chinese and Western Medicine. 2015; (4):373-4,81. doi:10.3969/j.issn.1008-8849.2015.04.010.

32. Wang HG, Yuan Y, Tang SW, Zheng YC, Li L. Clinical Study of Bailing Capsules Combined with Doxofylline in the Treatment of Chronic Obstructive Pulmonary Disease in the Elderly at Stable Stage. Evaluation and Analysis of Drug-Use in Hospitals of China. 2021; 21(3):295-8,305. doi:10.14009/j.issn.1672-2124.2021.03.010.

33. Ye YQ. Influences of bufei huoxue capsules in combination with tiotropium bromide on pulmonary function and exercise tolerance in patients with stable chronic obstructive pulmonary disease. Capital Medicine. 2020; 27(9):63-4. doi:10.3969/j.issn.1005-8257.2020.09.049.

34. Du SY, Chen HT. Observations on the efficacy and lung function of Fu Zheng Hua Tou Cream Formula for the treatment of chronic obstructive pulmonary disease in the stable stage of chronic obstructive pulmonary disease. Zhejiang Journal of Integrated Traditional Chinese and Western Medicine. 2015; 25(11):1044-5. doi:10.3969/j.issn.1005-4561.2015.11.026.

35. Fei XF, He Q, Xiao LN. Observation on the efficacy of Jin Gui Ren Qi Pill combined with Si Li Hua in treating patients with stable stage of COPD. Zhejiang Clinical Medical Journal. 2015; 17(10):1734-5.

36. Xia WJ, Mei XD, Wang XZ. Clinical Observation of Bufei Huoxue Capsules Combined with Azithromycin Tablets in the Treatment of Patients with Stable COPD. China Pharmacy. 2019; 30(10):1403-6. doi:10.6039/j.issn.1001-0408.2019.10.22.

37. Zhang H, Zhang Q, Liu MH. Clinical efficacy of bailing capsule in the treatment of chronic obstructive pulmonary disease. Chinese Journal of Clinical Rational Drug Use. 2016; 9(12):129-30.

38. Zhu DQ, Wu YR, Li QX. Clinical Observation of the Effect of Bufeihuoxue Capsule on Moderate and Severe Chronic Obstructive Pulmonary Disease (COPD). China Foreign Medical Treatment. 2013; 32(28):27-8.

39. Liu XW, Gao T, Chen JH. Effects of Su Huang Cough Capsules combined with tiotropium bromide on lung function and inflammatory factors in stable chronic obstructive pulmonary disease. Modern Traditional Chinese Medicine. 2018; 38(6):22-4. doi:10.13424/j.cnki.mtcm.2018.06.009.

40. Ma HX, Luo JJ. Effect of Yiqi Gubiao pill on Th17/Treg immune balance in peripheral blood of smoking patients with chronic obstructive pulmonary disease in stable stage. Hebei Journal of Traditional Chinese Medicine. 2018; 40(8):1146-50. doi:10.3969/j.issn.1002-2619.2018.08.006.

41. Song SL. Effect of Corbrin Capsule combined with routine western medicine on the airway remodeling process in patients with stable COPD. Journal of Hainan Medical University. 2017; 23(15):2033-6. doi:10.13210/j.cnki.jhmu.20170810.025.

42. Yang S. Efficacy of Bering capsule combined with budesonide formoterol powder inhaler in the treatment of stable COPD. Practical Clinical Medicine. 2019; 20(9):16-8. doi:10.13764/j.cnki.lcsy.2019.09.006.

43. Liu W, Xie YP, Fu H, Q. Effects of Jinshuibao capsule combined with budesonide formoterol on serum SP-D, HIF-1α and CXCL12 levels and cytokines in elderly patients with stable COPD. Chinese Journal of Gerontology. 2022; 42(5):1096-9. doi:10.3969/j.issn.1005-9202.2022.05.023.

44. Huang HT, Zhou Y, Liu XH, Sun PY, Zhang W. Effect of Feikang Granules on Pulmonary Function and Quality of Life in Patients with Stable Chronic Obstructive Pulmonary Disease. Journal of Guangzhou University of Traditional Chinese Medicine. 2019; 36(09):1305-11. doi:10.13359/j.cnki.gzxbtcm.2019.09.001.

45. Wang J, Yang B, Li L, Jiang LL, Le T, Tu XH*, et al.* Therapeutic efficacy and effect on peripheral blood Th17/Treg expression of Guoben Cough Granules combined with Sulidian in elderly patients with stable COPD Chinese Journal of Gerontology. 2022; 42(9):2131-4. doi:10.3969/j.issn.1005-9202.2022.09.026.

46. Yang LC, Chen JJ, Wu L. Clinical Study on Anchuan Zhisheng Ointment for Treatment of Chronic Obstructive Pulmonary Disease with Lung-kidney Yin Deficiency Syndrome. Chinese Journal of Information on Traditional Chinese Medicine. 2018; 25(8):21-4. doi:10.3969/j.issn.1005-5304.2018.08.006.

47. Xu T, Sun H. Clinical Observation of 170 Cases of Suhuangzhike Capsule in the Treatment of Stable Chronic Obstructive Pulmonary Disease. Chinese Journal of Medical Guide. 2015; 17(4):364-5. doi:10.3969/j.issn.1009-0959.2015.04.017.

48. Yang L, Li WC, Yang D, Zhu YF, Feng XL. Effect of Yifei Capsule and Umeclidinium Bromide and Vilanterol Powder for Inhalation on Lung Function,Cellular Immune Functio and Serum bFGF,SIRT1 in Patients with COPD at Stable Stage. Progress in Modern Biomedicine. 2023; 23(20):3908-12. doi:10.13241/j.cnki.pmb.2023.20.022.

49. Luo SW. Effects of Bailing capsule on lung function and activity of 51 cases in stable stage of COPD. Chinese Journal of Ethnomedicine and Ethnopharmacy. 2015; (2):34-,7. doi:10.3969/j.issn.1007-8517.2015.2.zgmzmjyyzz2015020018.

50. Song ZH, Xue J. Clinical study on Gejie Dingchuan Capsules combined with Salmeterol Xinafoate and Fluticasone Propionate Powder for inhalation in treatment of chronic obstructive pulmonary disease in the elderly at stable period. Drugs & Clinic. 2019; 34(7):2050-3. doi:10.7501/j.issn.1674-5515.2019.07.026.

51. Yang SW. The effect of Paring capsule combined with budesonide formoterol powder inhaler on pulmonary function and exercise tolerance in patients with chronic obstructive pulmonary disease in the stable stage of the disease. Chronic Pathematology Journal. 2021; (2):271-3.
